# Next generation haplotyping to decipher nuclear genomic interspecific admixture in *Citrus* species: analysis of chromosome 2

**DOI:** 10.1186/s12863-014-0152-1

**Published:** 2014-12-29

**Authors:** Franck Curk, Gema Ancillo, Andres Garcia-Lor, François Luro, Xavier Perrier, Jean-Pierre Jacquemoud-Collet, Luis Navarro, Patrick Ollitrault

**Affiliations:** UMR AGAP, Institut National de la Recherche Agronomique (Inra), Centre Inra de Corse, F-20230 San Giuliano, France; Centro de Protección Vegetal y Biotecnología, Instituto Valenciano de Investigaciones Agrarias (IVIA), 46113 Moncada, Valencia Spain; UMR AGAP, Centre de coopération Internationale en Recherche Agronomique pour le Développement (CIRAD), TA A-108/02, 34398 Montpellier, Cedex 5 France

**Keywords:** Phylogeny, Haplotype, Evolution, SNP, NGS, Genome admixture

## Abstract

**Background:**

The most economically important *Citrus* species originated by natural interspecific hybridization between four ancestral taxa (*Citrus reticulata*, *Citrus maxima*, *Citrus medica*, and *Citrus micrantha*) and from limited subsequent interspecific recombination as a result of apomixis and vegetative propagation. Such reticulate evolution coupled with vegetative propagation results in mosaic genomes with large chromosome fragments from the basic taxa in frequent interspecific heterozygosity. Modern breeding of these species is hampered by their complex heterozygous genomic structures that determine species phenotype and are broken by sexual hybridisation. Nevertheless, a large amount of diversity is present in the citrus gene pool, and breeding to allow inclusion of desirable traits is of paramount importance. However, the efficient mobilization of citrus biodiversity in innovative breeding schemes requires previous understanding of *Citrus* origins and genomic structures. Haplotyping of multiple gene fragments along the whole genome is a powerful approach to reveal the admixture genomic structure of current species and to resolve the evolutionary history of the gene pools. In this study, the efficiency of parallel sequencing with 454 methodology to decipher the hybrid structure of modern citrus species was assessed by analysis of 16 gene fragments on chromosome 2.

**Results:**

454 amplicon libraries were established using the Fluidigm array system for 48 genotypes and 16 gene fragments from chromosome 2. Haplotypes were established from the reads of each accession and phylogenetic analyses were performed using the haplotypic data for each gene fragment. The length of 454 reads and the level of differentiation between the ancestral taxa of modern citrus allowed efficient haplotype phylogenetic assignations for 12 of the 16 gene fragments. The analysis of the mixed genomic structure of modern species and cultivars (i) revealed *C. maxima* introgressions in modern mandarins, (ii) was consistent with previous hypotheses regarding the origin of secondary species, and (iii) provided a new picture of the evolution of chromosome 2.

**Conclusions:**

454 sequencing was an efficient strategy to establish haplotypes with significant phylogenetic assignations in *Citrus*, providing a new picture of the mixed structure on chromosome 2 in 48 citrus genotypes.

**Electronic supplementary material:**

The online version of this article (doi:10.1186/s12863-014-0152-1) contains supplementary material, which is available to authorized users.

## Background

World-wide production of citrus was 131 million tonnes in 2011 and 2012 [1]. The main citrus varietal groups are sweet oranges (52%), small citrus (21%), limes and lemons (12%), and grapefruits and pummelos (6%). The inter-varietal genetic diversity of most of these varietal groups is very scarce, particularly for sweet oranges, lemons, and grapefruits, where intra-group diversity results from clonal variation/selection in vegetatively propagated material [2]. This confers a substantial fragility of these groups against emerging diseases, as demonstrated by the ongoing major crisis in the Brazilian and Floridian citrus industries [3-5]. Moreover, conventional breeding of these species is hampered by their complex heterozygous genomic structures that determine species phenotype and are broken by sexual hybridisation. Therefore, most breeding efforts for sweet orange, grapefruit, and lemons to date have used natural or induced mutations and somaclonal variation [6]. However, important natural phenotypically useful variability exists in the citrus gene pool particularly for resistance to biotic and abiotic constraints [7]. The efficient mobilization of this biodiversity in innovative breeding schemes will require prior knowledge of varietal group origins and genomic structures.

The taxonomy of *Citrus* remains controversial due to the conjunction of broad morphological diversity, total interspecific sexual compatibility within the genus, and partial apomixis of many cultivars. Fixing complex genetic structures through seedling propagation via apomixis led some taxonomists to consider clonal families of interspecific origin as new species [8]. Two major systems are widely used to classify *Citrus* species: the Swingle and Reece [9] classification, which identifies 16 species, and the Tanaka [10] classification, which recognizes 156 species. More recently, Mabberley [11] proposed a new classification of edible citrus that recognized three species and four hybrid groups. In this paper, we will use the Swingle and Reece [9] classification system. This taxonomic system is widely used in the citrus scientific community and, as mentioned below, mostly agrees with molecular data. Despite the difficulties involved in establishing a consensus classification system for edible citrus, most authors now agree on the origins of the main cultivated forms. Molecular analyses clarified the genetic underpinnings of various cultivated species of *Citrus* [12-18]. Four ancestral taxa [*C. medica* L. (citron), *C. reticulata* Blanco (mandarin), *C. maxima* (Burm.) Merr. (pummelo), and *C. micrantha* Wester (papeda)] were identified as the ancestors of all cultivated *Citrus* [13,15]. Differentiation between these sexually compatible taxa may be explained by foundation effects in three distinct geographic zones and by an initial allopatric evolution. *C. maxima* originated in the Malay Archipelago and Indonesia, *C. medica* evolved in northeastern India and the nearby region of Myanmar and China, and *C. reticulata* diversification occurred over a region including Vietnam, southern China, and Japan [8,19]. Secondary species [*C. sinensis* (L.) Osb. (sweet orange), *C. aurantium* L. (sour orange), *C. paradisi* Macf. (grapefruit), *C. limon* (L.) Burm. (lemon), and *C. aurantifolia* (Christm.) Swing. (lime)] arose from hybridizations between the four basic taxa [13,15]. Partial apomixis of most of the secondary species has been an essential element in the limitation of the number of further interspecific meiosis. Moreover, studies considering diversity of morphological characteristics [20,21], primary metabolites [22], and secondary metabolites [23] confirmed that a major part of the phenotypic diversity of edible citrus resulted from differentiation between the basic taxa. In this context, deciphering the phylogenomic structures of the secondary citrus species is essential before innovative conventional breeding strategies can be developed.

Reticulations pose serious challenges in phylogenetic analyses and result in evolutionary histories that cannot be adequately represented in the form of phylogenetic trees [24-28]. For many species, these relationships resemble a network with phylogenetic incongruities observed not only between cytoplasmic and nuclear genomes, but also between different regions of nuclear genomes [29-32]. In plants such as citrus, where vegetative propagation such as apomixis took place immediately or a few generations after a reticulation event, large parts of the genome remain in interspecific heterozygosity. Genome-wide molecular analyses are, therefore, needed to decipher the complex interspecific mosaic genomes resulting from such evolution. Studies based on linkage disequilibrium can provide good evidence for recent and ancient hybridization events. This was demonstrated in sunflower by Rieseberg *et al*. [33,34], who showed that the genomes of hybrid sunflower species contained chromosomal segments from both parental species. When examining heterozygous structures like citrus genotypes, phased multilocus studies offer improvements over monolocus analysis for the identification of interspecific heterozygous genome fragments deriving from reticulate events. The expectation is that tightly linked markers in a hybrid species are significantly more likely to come from the same parent and, therefore, to display linkage disequilibrium [29]. Sanger sequencing after bacterial cloning to separate gene copies was used effectively for such analysis [35-37]. However, because this is time-consuming and expensive, and only a few individuals and genes can be investigated, this type of analysis can miss intraspecific diversity components and may lead to erroneous conclusions about the evolutionary history of related taxa [38]. In recent years, massively parallel sequencing of barcoded DNA mixtures enabled rapid and relatively inexpensive DNA sequence data production and facilitated genome-wide sequence variant discovery. This analysis was applied to a wide variety of bacteria, fungi [39,40], multi-copy genes [41], and polyploids. In citrus, recent whole genome sequencing projects [42,43] confirmed hybridization at the origin of *C. sinensis* and *C. clementina* (clementine) and allowed the phylogenetic origin of DNA fragments in the whole genome to be determined. However, the genomic structure of other secondary species and most modern varieties remain to be studied, and no analysis of the phylogeny of DNA fragments from the whole genome has yet been undertaken.

Whole genome sequencing (WGS) in large populations remains costly and requires considerable bioinformatic analysis. Major challenges include the need to reduce genome complexity and manage orthologous sequence data for a large number of individuals. Alternatives such as targeted capture [44] or targeted amplicon [45] sequencing can be valuable. In human research, deep amplicon sequencing using 454 technology yielded thousands of haplotype calls per amplicon at the beta-defensin locus, and this was considered to be an efficient method for haplotyping and copy-number estimation in small to medium-sized cohorts [41]. A particular advantage of using such an approach for haplotyping heterozygous structures is that sequencing data come from single DNA molecules, and there is no requirement for cloning. Therefore, we hypothesize that, by using a sequencing method allowing enough long reads (over 500 bp) such as 454 pyrosequencing [41], it should be possible to establish multilocus haplotypes that are phylogenetically significant when working at a sufficient level of genetic differentiation between taxa.

The objective of this work was to analyze the potential of the 454 sequencing method for efficient targeted parallel haplotyping to decipher complex interspecific genomic structures resulting from reticulate evolution in citrus. Amplicons from 48 genotypes, representative of *Citrus* ancestral taxa and secondary species, were subjected to parallel sequencing. Sixteen targeted genes distributed across chromosome 2 were sequenced. Chromosome 2 was selected due to its complex admixture structure in sweet orange, as identified in our previous research [16,43].

## Methods

### Plant material

Leaves from 48 accessions of the *Citrus* genus and one accession of *Severinia buxifolia* [Poir.] Tenore were collected from the IVIA Citrus Germplasm Bank of pathogen-free plants (Valencia, Spain; accessions with IVIA identification number) and the INRA/CIRAD Citrus collection of San Giuliano (Corsica, France; accessions with SRA identification number) [Additional file 1]. In addition, in silico data were mined (phytozome.net [46]) from the haploid clementine used to establish the first high-quality reference sequence of Citrus [43].

The Swingle and Reece [9] botanical classification for scientific names was adopted (Table 1 and [Additional file 1]). The four ancestral taxa of the *Citrus* genus were represented by 31 accessions: 14 mandarins (12 *C. reticulata* and two *C. tachibana* (Mak*.*) Tan.), ten pummelos (*C. maxima*), six citrons (*C. medica*), and one papeda (*C. micrantha*). Representatives of secondary citrus species or genotypes included two diploid clementines (*C. reticulata*), the haploid clementine used to establish the whole citrus genome reference sequence (*C. reticulata*), three sweet oranges (*C. sinensis*), two sour oranges (*C. aurantium*), two grapefruits (*C. paradisi*), five lemons (*C. limon*), one bergamot (*C. aurantifolia*), one lime (*C. aurantifolia*), and one ‘Alemow’ (*C. aurantifolia*). These 18 genotypes were putative hybrids derived from the four ancestral taxa. One *Citrus* genus relative (*Severinia buxifolia*) was added as an out-group.

**Table 1:** **Scientific names and number of accessions per common horticultural group**

	**Common horticultural group name**	**Swingle scientific name**	**Number of accessions**
Ancestral groups	Pummelo	*Citrus maxima* (Burm.) Merr.	10
	Mandarin	*Citrus reticulata* Blanco	12
		*Citrus tachibana* (Mak.) Tan.	2
	Citron	*Citrus medica* L.	6
	Papeda	*Citrus micrantha* Wester	1
Secondary species or genotypes arising from hybridizations between ancestral groups	Bergamot	*Citrus aurantifolia* (Christm.) Swing.	1
	Lime	*Citrus aurantifolia* (Christm.) Swing.	1
	Alemow	*Citrus aurantifolia* (Christm.) Swing.	1
	Sour orange	*Citrus aurantium* L.	2
	Lemon	*Citrus limon* (L.) Burm.	5
	Grapefruit	*Citrus paradisi* Macf.	2
	Clementine	*Citrus reticulata* Blanco	3
	Sweet orange	*Citrus sinensis* (L.) Osb.	3
Out-group		*Severinia buxifolia* (Poir.) Ten.	1

### DNA extraction

High molecular weight genomic DNA was extracted from leaf samples using the DNeasy Plant Mini Kit (Qiagen S.A.; Madrid, Spain) according to the manufacturer’s instructions.

### Target genomic fragment selection

#### Chromosome 2 targeted genomic fragments

The reference citrus whole genome sequence, released in Phytozome [46] by the International Citrus Genome Consortium (ICGC), was used to select gene fragments in this study. The annotated genes file (“Cclementina_182_gene.gff3” file) was used and is available at the Phytozome web page [46].

Duplicated and overlapping genes were discarded. SSRs were annotated (up to tetranucleotidic motifs and at least 11 bp sequences) and all genes presenting microsatellite motifs were eliminated. Finally, the genes were sorted by length, and 415 genes were selected, each with a length of 1000–2000 bp. This length was selected to facilitate the design of primers for efficient sequencing of 500–600 bp amplicons. Sixteen genes within chromosome 2 were chosen.

### Amplicon library preparation

For the 16 selected gene fragments of chromosome 2 [Additional file 2], 16 primer pairs were designed (according to the Access Array™ System for 454 Sequencing Platform User Guide [47]) and loaded on the Fluidigm Access Array. This method employed the same approach as the two-step PCR methods proposed by Bybee *et al*. [45] and validated by Curk *et al.* [48] for citrus. Two successive PCR reactions produced amplicons with specific multiplex identifiers (MIDs) and directional titanium primer sequences for each variety. PCR products were generated using a 48.48 Access Array IFC (Fluidigm 48.770 Digital PCR Workflow Quick Reference Card), and amplicon quality was checked using an Agilent 2100 Bioanalyzer (Agilent DNA 1000 Kit Guide). Next, equal volumes of the PCR products were pooled together to create one PCR product library. The PCR product library was purified using AMPure beads. After purification, the PCR product library was quantified using Quant-iT PicoGreen fluorimetry (Quant-iT™ PicoGreen® User Guide) before proceeding to emulsion PCR.

454 parallel sequencing was performed using a mixture of all the amplicons for all the genotypes. DNA from each genotype carried a different MID, as defined by Roche [49]. The 454 sequencing technique requires amplicon primers to contain a directional GS FLX Titanium primer sequence (which includes a four base library “key” sequence) at the 5′ end of the oligonucleotide in addition to the gene-specific sequence at the 3′ end. To allow for automated software identification of samples after pooling and sequencing, MID sequences [Additional file 3] were added between primer A (or B) and the gene-specific sequences [50].

Forty-eight DNA samples were amplified and parallel-sequenced on a GS FLX Titanium system (Roche 454). Haploid clementine gene fragment sequences were obtained from the reference citrus whole genome sequence (Phytozome [46]). *S. buxifolia* (out-group) gene fragments were obtained by PCR amplification performed using a Mastercycler Ep gradient S thermocycler (Eppendorf). PCR was conducted in a final volume of 25 μl containing 0.027 U Taq DNA polymerase (Fermentas), 1 ng/μl of genomic DNA, 10 × PCR buffer (Fermentas), 0.2 mM of each dNTP, 1.5 mM MgSO_4_, and 0.2 μM of each primer. The following PCR program was applied: denaturation at 94°C for 5 min; 40 cycles of 30 s at 94°C, 1 min at 55°C, and 2 min at 72°C; and a final elongation step of 4 min at 72°C. PCR product purification was performed using a QIAquick PCR purification kit (Qiagen S.A.). Amplicons were sequenced using the Sanger method from the 5′ end using fluorescently labeled dideoxynucleotides (Big Dye Terminator Cycle Sequencing Kit v3.1).

### Sequencing and sequence data analysis for SNP calling

Raw reads obtained from 454 pyrosequencing were preprocessed by removal of low-quality reads and adapter/primer sequences using PRINSEQ [51]. Short reads (<150 bases) with primer dimers were considered to be low-quality reads. Remaining reads were automatically identified and sorted by MID and specific gene primers using the SFF Tool commands of Newbler software [52].

For each variety, 454 pyrosequencing reads were aligned independently for each gene using SeqMan NGen software version 7.0 [53] with the following assembly parameters: match size, 12; minimum match percentage, 80; and minimum sequence length, 150. For each gene fragment, consensus alignments from a homozygous sequence comprised one haplotype, while those from a heterozygous sequence comprised two haplotypes.

### Genetic analysis of SNP data

Unbiased expected heterozygosity (He), observed heterozygosity (Ho), fixation index values (F_W_ [54]), and F_Stat_ parameters (F_ST_ and G_ST_ Index) were calculated using GENETIX v. 4.03 software [55]. SNP number and location were identified with SNiPlay online software [56,57]. Principal component analysis (PCA) was performed using XLSTAT software.

Haplotype and genotypic phylogenetic relationships were studied by (i) neighbor-joining analysis (NJA), based on the SNP data using DARwin software [58] with a simple matching dissimilarity index, (ii) maximum likelihood phylogenetic analysis using Mega software [59]. The simple matching dissimilarity index was also used to infer intra- and inter-taxa average differentiation.

Graphical visualization of chromosome 2 genotypes was constructed using GGT2 software [60].

Population structure was inferred using Structure (version 2.3.4) software [61], which implements a model-based clustering method using genotype data [62,63]. No a priori population structure was defined. The linkage model option was used, with allele frequencies correlated and compute probability of the data for estimating K. Analyses were made with K-values (number of subpopulations) of 1–10. The statistics used to select the correct K-value were those used by Evanno *et al.* [64]. Ten runs using Structure software were performed, each with 50,000 steps of burning followed by 50,000 Monte Carlo Markov Chain (MCMC) repetitions using the linkage model, knowing Map distances between loci [Additional file 2] [17]. The independent Structure-run cluster outputs were permuted and aligned to match one another as closely as possible.

## Results

### Read distribution

**Figure 1:**
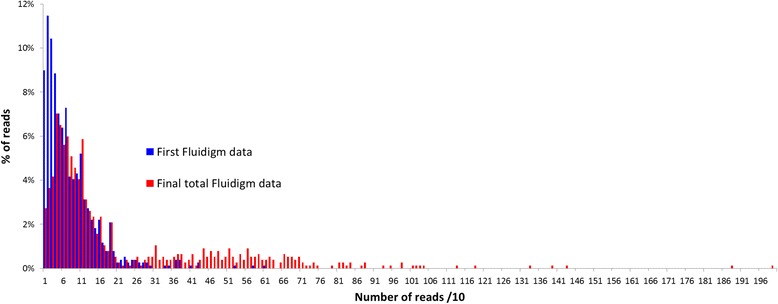
**Distribution of the numbers of reads per amplicon for two rounds of Fluidigm/454 sequencing.**

The first round of Fluidigm amplification/454 sequencing produced 64,170 reads. Of these, 11% were short reads with primer dimers, and 57,394 reads were therefore considered useful. Useful reads were classified according to their MID and titanium sequences, and MID sequences were removed using 454 software tools. All reads were attributed to one of the 768 (48 × 16) amplicons according to the fragment gene sequence. The average number of reads per amplicon was 75; however, the distribution of reads per amplicon (Figure 1) was asymmetric, resulting in a high proportion of amplicons with insufficient coverage. Based on 454 single-read sequencing data error rates and our preliminary unpublished data, we defined a threshold level of 50 reads per amplicon for confident genotype calling. However, 305 amplicons (40%) had fewer than this initial threshold number. Detailed analysis of read distribution for each amplicon [Additional file 4] showed that much of the heterogeneity was due to global under-representation of three gene fragments and over-representation of five fragments. The total number of reads per variety was less heterogeneous than one per gene fragment. We therefore conducted a second round of Fluidigm/454 sequencing. A total of 159,490 useful reads was obtained (average 208 reads per amplicon) from the combination of the two runs [Additional file 5]. The distribution of the number of reads per amplicon remained highly heterogeneous, and 135 amplicons (18% of the total gene fragments/varieties) still had fewer than 50 associated reads. In cases where number or quality of reads was insufficient for genotype calling, amplicons were Sanger sequenced to complete the genotypic data set. Sanger sequence analysis also allowed inference of haplotype if only one or no heterozygous loci were observed in the Sanger sequence [Additional file 5].

### Genotype calling and polymorphism of gene fragments

A total of 318 SNPs were identified from 7895 bp readable sequences for the 16 gene fragments within the 48 *Citrus* accessions (Table 2). The web based SNiPlay tool [56,57] was used to analyze the intragenic location and potential impact of the different SNPs according to the whole genome annotation available at phytozome.net. The vast majority (98%) of the SNP loci was diallelic, but 2% (seven loci) were triallelic (Table 2). The tri-allelism was validated by Sanger sequencing (data not shown). Sanger sequencing of the 2P33506778 fragment was performed for 32 *Citrus* varieties to estimate the 454 SNP-calling error rate. Only three differences between 454 and Sanger data were observed over 17,152 bp genotyping data (32 genotypes per 536 bp fragment; 0.02% error rate). The ‘Clemenules’ clementine was homozygous according to Sanger sequencing, but had two heterozygotic SNPs according to the 454 sequencing data. The ‘Beauty’ mandarin was shown to be heterozygous with the two techniques, but one of the three heterozygotic 454 SNPs was not identified in the Sanger data. The average SNP frequencies in intronic, exonic and 3′ UTR regions were 53.57, 38.77, and 39.77 SNPs/kb, respectively. In addition, five indels were found in exonic regions (fragments 2P8108334, 2P26819388, and 2P32507721 contained one indel, and 2P29538734 contained two).

**Table 2:** **SNP number and location for 16 gene fragments sequenced in 48 diploid** ***Citrus*** **genotypes**

**Gene fragment**	**Total Sequence**				**Intron**			**Exon**			**3′-UTR**		
	**Seq size**	**SNP**	**SNP/kb**	**Trialelic SNP**	**Seq size**	**SNP**	**SNP/kb**	**Seq size**	**SNP**	**SNP/kb**	**Seq size**	**SNP**	**SNP/kb**
2P737170	452	22	48.67	Exon	0	0	-	452	22	48.67	0	0	-
2P3068140	421	14	33.25	_	337	12	35.61	84	2	23.81	0	0	-
2P4517048	502	12	23.90	_	0	0	-	316	4	12.66	186	8	43.01
2P8108334	502	40	79.68	Exon	0	0	-	502	40	79.68	0	0	-
2P11442721	547	21	38.39	Exon	0	0	-	547	21	38.39	0	0	-
2P13928427	502	21	41.83	_	0	0	-	336	15	44.64	166	6	36.14
2P21022460	538	11	20.45	_	0	0	-	538	11	20.45	0	0	-
2P25198627	454	12	26.43	_	128	7	54.69	326	5	15.34	0	0	-
2P26819388	535	22	41.12	Exon	0	0	-	535	22	41.12	0	0	-
2P29538734	541	36	66.54	Exon	190	12	63.16	351	24	68.38	0	0	-
2P30446231	475	28	58.95	_	216	15	69.44	259	13	50.19	0	0	-
2P32507721	463	16	34.56	_	0	0	-	463	16	34.56	0	0	-
2P33532337	459	9	19.61	_	0	0	-	459	9	19.61	0	0	-
2P33506778	536	6	11.19	Exon	0	0	-	536	6	11.19	0	0	-
2P35391362	449	19	42.32	_	108	6	55.56	341	13	38.12	0	0	-
2P36235952	519	29	55.88	Exon	141	8	56.74	378	21	55.56	0	0	-
**16**	**7895**	**318**	**40.28**		**1120**	**60**	**53.57**	**6423**	**244**	**37.99**	**352**	**14**	**39.77**

### SNP diversity differentiation

Previous molecular studies [14,16,43] showed that some varieties of the main *Citrus* cultivar groups had interspecific introgressions. Therefore, in this study, we differentiated mandarin, pummelo, and citron groups of their respective pure ancestral taxa: *C. reticulata*, *C. maxima*, and *C. medica*.

For genotypic based analyses, we refer to the modern varietal groups, while we focus on pure ancestral taxa for the haplotype phylogenetic analyses.

Only 19 of the 318 SNPs were not found in the accessions representing the four basic taxa. These rare alleles were identified in heterozygosity in secondary species (‘Alemow’, nine; sour oranges, four; bergamot, three, ‘Volkamer’ lemon, one; ‘Mexican’ lime, one; and grapefruit, one) and concerned 9 of the 16 gene fragments. The parameters of SNP genetic diversity given in Table 3 (and detailed in [Additional file 6] for each SNP position) were calculated without these 19 rare alleles. The whole population displayed a diversity index (He) of 0.23 and a fixation index (F_W_) value of 0.29, suggesting an important population genetic structure of the analysed varietal sample. Mandarin and pummelo intra-diversity F_W_ values were close to zero, but intra-group polymorphism was higher in mandarin (He = 0.12 ± 0.02) than in pummelo (He = 0.07 ± 0.02). Citron displayed low heterozygosity (Ho = 0.02 ± 0.01) and diversity (He = 0.03 ± 0.01). Only one *C. micrantha* representative was available: the observed heterozygosity value (0.09; ± 0.09) was, therefore, calculated between the pummelo and mandarin values. The average numbers of SNPs/kb between two varieties within and between the four supposed basic taxa were 1.26–3.93 SNPs/kb within groups and 10.41–14.56 SNPs/kb at the inter-group level (Table 4).

**Table 3:** **SNP genetic diversity within and between supposed ancestral varietal groups**

	**Whole population**			**Citrons**			**Mandarins**			***Citrus micrantha***			**Pummelos**			**4 populations**
	**Ho**	**He**	**F**_**W**_	**Ho**	**He**	**F**_**W**_	**Ho**	**He**	**F**_**W**_	**Ho**	**He**	**F**_**W**_	**Ho**	**He**	**F**_**W**_	**F**_**ST**_
2P737170	0.11	0.23	0.52	0.02	0.03	0.33	0.01	0.01	−0.04	0.15	0.08	−1.00	0.08	0.07	−0.22	0.78
SD	0.07	0.15	0.25	0.02	0.11	0.00	0.03	0.02	0.00	0.36	0.18	0.00	0.04	0.15	0.08	0.33
CI	0.03	0.07	0.11	0.02	0.05	-	0.01	0.01	-	0.16	0.08	-	0.02	0.07	0.08	0.14
2P3068140	0.18	0.33	0.46	0.00	0.00	-	0.07	0.10	−0.01	0.00	0.00	-	0.02	0.02	−0.18	0.72
SD	0.08	0.17	0.22	0.00	0.00	-	0.12	0.10	0.13	0.00	0.00	-	0.04	0.07	-	0.40
CI	0.05	0.09	0.12	-	-	-	0.06	0.05	0.07	-	-	-	0.02	0.04	-	0.22
2P4517048	0.09	0.19	0.55	0.01	0.01	−0.09	0.10	0.08	−0.17	0.00	0.00	-	0.06	0.05	−0.14	0.48
SD	0.07	0.19	0.33	0.03	0.04	-	0.07	0.18	0.22	0.00	0.00	-	0.08	0.09	0.04	0.47
CI	0.04	0.11	0.19	0.02	0.02	-	0.04	0.10	0.25	-	-	-	0.05	0.05	0.04	0.27
2P8108334	0.12	0.20	0.42	0.01	0.04	0.80	0.04	0.06	0.38	0.21	0.10	−1.00	0.14	0.09	−0.52	0.52
SD	0.09	0.18	0.36	0.01	0.10	0.49	0.05	0.14	0.28	0.41	0.20	0.00	0.06	0.17	0.33	0.36
CI	0.03	0.06	0.11	0.01	0.03	0.39	0.03	0.04	0.18	0.13	0.06	-	0.04	0.05	0.20	0.11
2P11442721	0.15	0.18	0.18	0.02	0.02	−0.09	0.23	0.20	−0.19	0.00	0.00	-	0.06	0.06	0.04	0.32
SD	0.11	0.13	0.26	0.05	0.05	0.00	0.09	0.20	0.16	0.00	0.00	-	0.06	0.12	0.32	0.35
CI	0.05	0.05	0.11	0.04	0.02	0.00	0.05	0.08	0.09	-	-	-	0.04	0.05	0.28	0.15
2P13928427	0.11	0.17	0.38	0.04	0.05	0.17	0.06	0.05	−0.11	0.10	0.05	−1.00	0.01	0.01	−0.05	0.40
SD	0.08	0.16	0.23	0.04	0.15	0.00	0.11	0.08	0.05	0.30	0.15	0.00	0.03	0.03	0.00	0.38
CI	0.04	0.07	0.10	0.04	0.06	-	0.06	0.04	0.04	0.13	0.07	-	0.02	0.01	-	0.16
2P21022460	0.12	0.19	0.34	0.00	0.04	1.00	0.05	0.06	0.11	0.18	0.09	−1.00	0.10	0.07	−0.50	0.49
SD	0.08	0.13	0.28	0.00	0.13	-	0.07	0.13	0.11	0.39	0.20	0.00	0.03	0.16	0.35	0.41
CI	0.05	0.08	0.16	-	0.08	-	0.03	0.08	0.11	0.23	0.12	-	0.02	0.09	0.48	0.24
2P25198627	0.17	0.21	0.20	0.08	0.10	0.16	0.10	0.13	0.23	0.17	0.08	−1.00	0.20	0.15	−0.38	0.34
SD	0.14	0.16	0.20	0.08	0.18	0.61	0.14	0.18	0.22	0.37	0.19	0.00	0.10	0.22	0.05	0.33
CI	0.08	0.09	0.12	0.06	0.10	0.69	0.07	0.10	0.15	0.21	0.11	-	0.06	0.12	0.05	0.19
2P26819388	0.09	0.16	0.46	0.07	0.05	−0.33	0.08	0.13	0.45	0.32	0.17	−1.00	0.02	0.02	−0.33	0.25
SD	0.10	0.17	0.30	0.06	0.13	0.00	0.10	0.19	0.36	0.47	0.24	0.00	0.02	0.08	-	0.18
CI	0.04	0.07	0.12	0.05	0.05	-	0.07	0.08	0.21	0.19	0.10	-	0.01	0.03	-	0.07
2P29538734	0.17	0.26	0.34	0.00	0.03	1.00	0.19	0.16	−0.18	0.06	0.03	−1.00	0.05	0.05	−0.07	0.53
SD	0.11	0.16	0.28	0.00	0.09	0.00	0.16	0.17	0.09	0.23	0.11	0.00	0.06	0.10	0.37	0.39
CI	0.04	0.05	0.09	-	0.03	-	0.08	0.05	0.04	0.08	0.04	-	0.04	0.03	0.23	0.13
2P30446231	0.12	0.20	0.37	0.02	0.04	0.56	0.10	0.11	0.07	0.08	0.04	−1.00	0.10	0.16	0.39	0.47
SD	0.09	0.14	0.28	0.04	0.12	0.00	0.12	0.17	0.11	0.27	0.14	0.00	0.07	0.20	0.44	0.37
CI	0.03	0.05	0.11	0.03	0.05	-	0.06	0.07	0.07	0.10	0.05	-	0.05	0.08	0.26	0.14
2P32507721	0.14	0.21	0.31	0.00	0.00	-	0.29	0.31	0.06	0.18	0.08	−1.00	0.07	0.04	−0.60	0.43
SD	0.10	0.14	0.18	0.00	0.00	-	0.25	0.24	0.05	0.39	0.19	0	0.04	0.14	-	0.32
CI	0.06	0.08	0.10	-	-	-	0.13	0.14	0.03	0.23	0.11	-	0.02	0.08	-	0.18
2P33506778	0.18	0.29	0.37	0.00	0.00	-	0.18	0.17	−0.07	0.00	0.00	-	0.05	0.06	0.20	0.81
SD	0.13	0.17	0.11	0.00	0.00	-	0.17	0.21	0.02	0.00	0.00	-	0.08	0.15	-	0.20
CI	0.10	0.13	0.09	-	-	-	0.09	0.17	0.03	-	-	-	0.05	0.12	-	0.16
2P33532337	0.19	0.32	0.39	0.00	0.07	1.00	0.23	0.24	−0.14	0.00	0.00	-	0.04	0.09	0.52	0.65
SD	0.11	0.15	0.15	0.00	0.08	0.00	0.26	0.17	0.20	0.00	0.00	-	0.09	0.19	0.00	0.31
CI	0.07	0.10	0.10	-	0.05	-	0.14	0.11	0.15	-	-	-	0.06	0.12	-	0.21
2P35391362	0.22	0.37	0.41	0.01	0.01	−0.09	0.24	0.20	−0.24	0.00	0.00	-	0.02	0.06	−0.15	0.73
SD	0.09	0.13	0.16	0.02	0.04	-	0.24	0.18	0.10	0.00	0.00	-	0.03	0.09	0.09	0.24
CI	0.04	0.06	0.07	0.02	0.02	-	0.13	0.08	0.06	-	-	-	0.02	0.04	0.06	0.11
2P36235952	0.13	0.28	0.55	0.00	0.00	-	0.02	0.04	0.45	0.04	0.02	−1.00	0.08	0.07	−0.28	0.55
SD	0.08	0.21	0.34	0.00	0.00	-	0.04	0.11	0.35	0.20	0.10	0.00	0.02	0.13	0.15	0.47
CI	0.03	0.08	0.14	-	-	-	0.02	0.04	0.26	0.08	0.04	-	0.01	0.05	0.12	0.19
Total	0.14	0.23	0.29	0.02	0.03	0.46	0.12	0.12	−0.02	0.09	0.05	−1.00	0.07	0.07	−0.08	0.51
SD	0.10	0.17	0.28	0.01	0.10	0.53	0.05	0.17	0.24	0.19	0.15	0.00	0.02	0.14	0.37	0.38
CI	0.03	0.02	0.03	0.01	0.01	0.19	0.03	0.02	0.04	0.09	0.02	-	0.01	0.02	0.09	0.04

Ho: observed heterozygosity; He: expected heterozygosity; F_W_: fixation index; F_ST_: fixation index within population; SD: standard deviation; CI: confidence interval estimated with alpha = 0.05.

**Table 4:** **Intra- and inter-varietal group dissimilarities (average number of SNP/kb between two varieties)**

	**Mandarins**	**Pummelos**	**Citrons**
Mandarins	3.93^*^		
Pummelos	10.41	2.06^*^	
Citrons	14.56	11.21	1.26^*^
*C. micrantha*	13.49	10.61	12.24

*Average number of SNP/kb at intra-specific level.

For secondary species, no intraspecific polymorphism was observed for sweet oranges, grapefruits, and sour oranges, represented, respectively, by three, two, and two varieties. The two clementine cultivars were also found to be identical. Polymorphism was found between regular lemons and the other ones; however, the two regular lemons (‘Eureka’ and ‘Lisbon’) and ‘Sweet’ lemon were found to be identical. Acid citrus types (lemons, limes, ‘Alemow’, and bergamot) and sour orange displayed high Ho values (0.26–0.34 ± 0.05). Sweet orange (0.15 ± 0.04), clementine (0.19 ± 0.04), and grapefruit (0.12 ± 0.04) displayed comparatively lower heterozygosity levels [Additional file 7].

**Figure 2:**
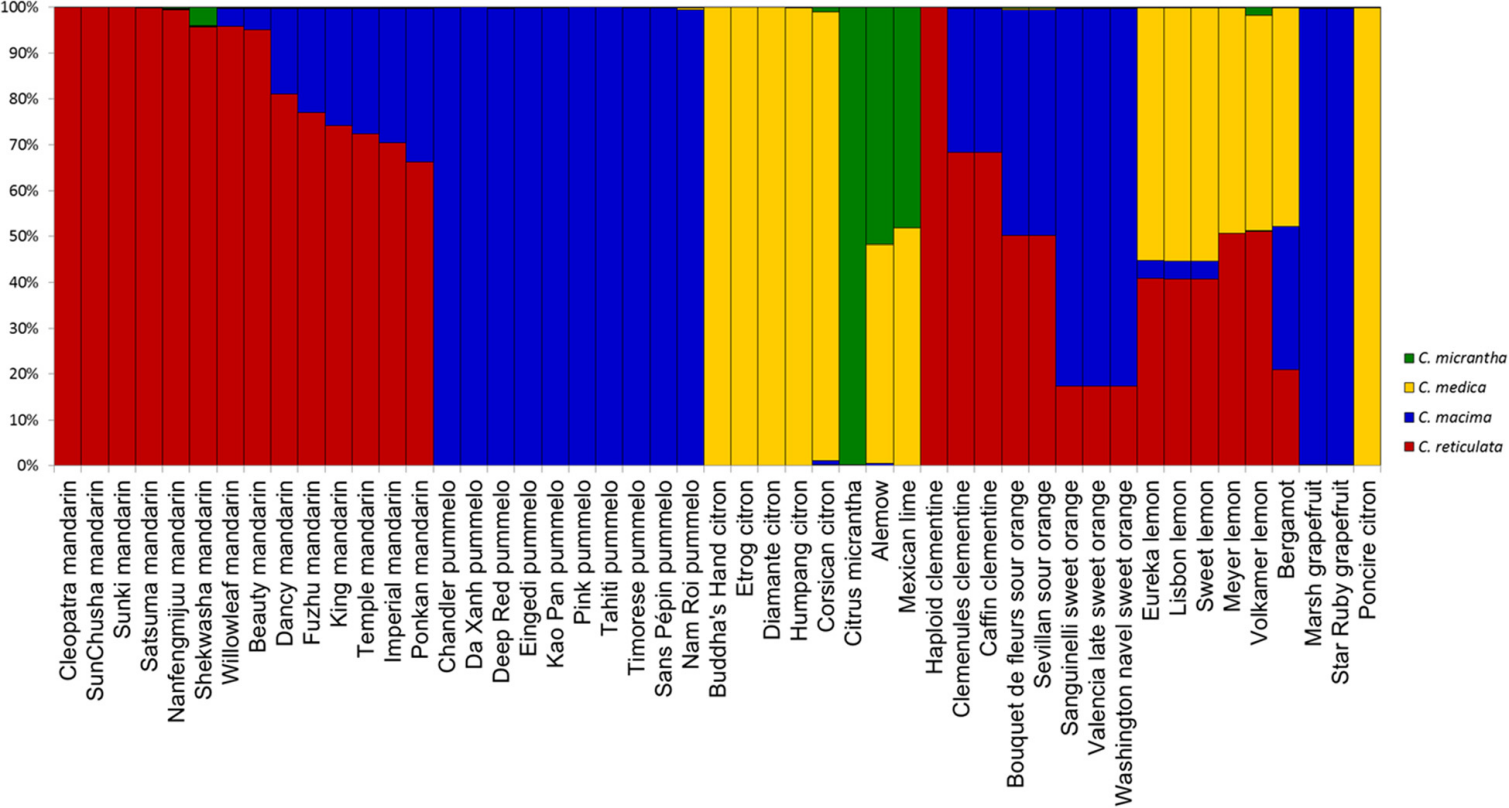
**Estimated population structure representation based on the average values of ten Structure runs at K = 4.**

Structure software analysis was performed in the absence of a prior hypothesis for group number. Analysis of ΔK identified K = 4 as the optimal population number. The ten runs for K = 4 displayed very homogeneous results (as shown by the average values [Figure 2, Additional file 8]). *C. medica*, *C. maxima*, and *C. micrantha* defined three populations, and five mandarins defined a fourth population. The magnitude of genetic differentiation between the groups was statistically confirmed by the pairwise F_ST_ values, which ranged from 0.499 ± 0.091 for *C. maxima*/*C. micrantha* to 0.719 ± 0.087 for C. *micrantha*/C. *medica* (Table 5). Eight of the additional mandarins appeared to belong chiefly to this last group but exhibited introgression from the *C. maxima* group. ‘Shekwasha’ mandarin displayed a possible introgression of *C. micrantha*. Some cultivars displayed more pronounced genetic mixing. ‘Alemow’ and ‘Mexican’ lime had half their features from the *C. micrantha* group and half from the *C. medica* group. Similarly, sour oranges had half their features from each of the *C. reticulata* and *C. maxima* groups. Sweet orange and clementine were admixtures of the *C. maxima* and *C. reticulata* groups.. Regular and ‘Sweet’ lemons and bergamot were admixtures of three groups: *C. maxima*, *C. reticulata*, and *C. medica*. Close to half of the genetic material in ‘Volkamer’ and ‘Meyer’ lemons was of the *C. medica* group, and half was of the *C. reticulata* group [Figure 2, Additional file 8].

**Table 5:** **Pairwise population F**_**ST**_ **values**

	**Mandarin**		**Pummelo**		**Citron**	
	**SNP**	**F**_**ST**_	**SNP**	**F**_**ST**_	**SNP**	**F**_**ST**_
Pummelo	172	0.502 ± 0.061				
Citron	171	0.666 ± 0.061	142	0.585 ± 0.066		
*C. micrantha*	167	0.574 ± 0.079	143	0.499 ± 0.091	127	0.719 ± 0.087

**Figure 3:**
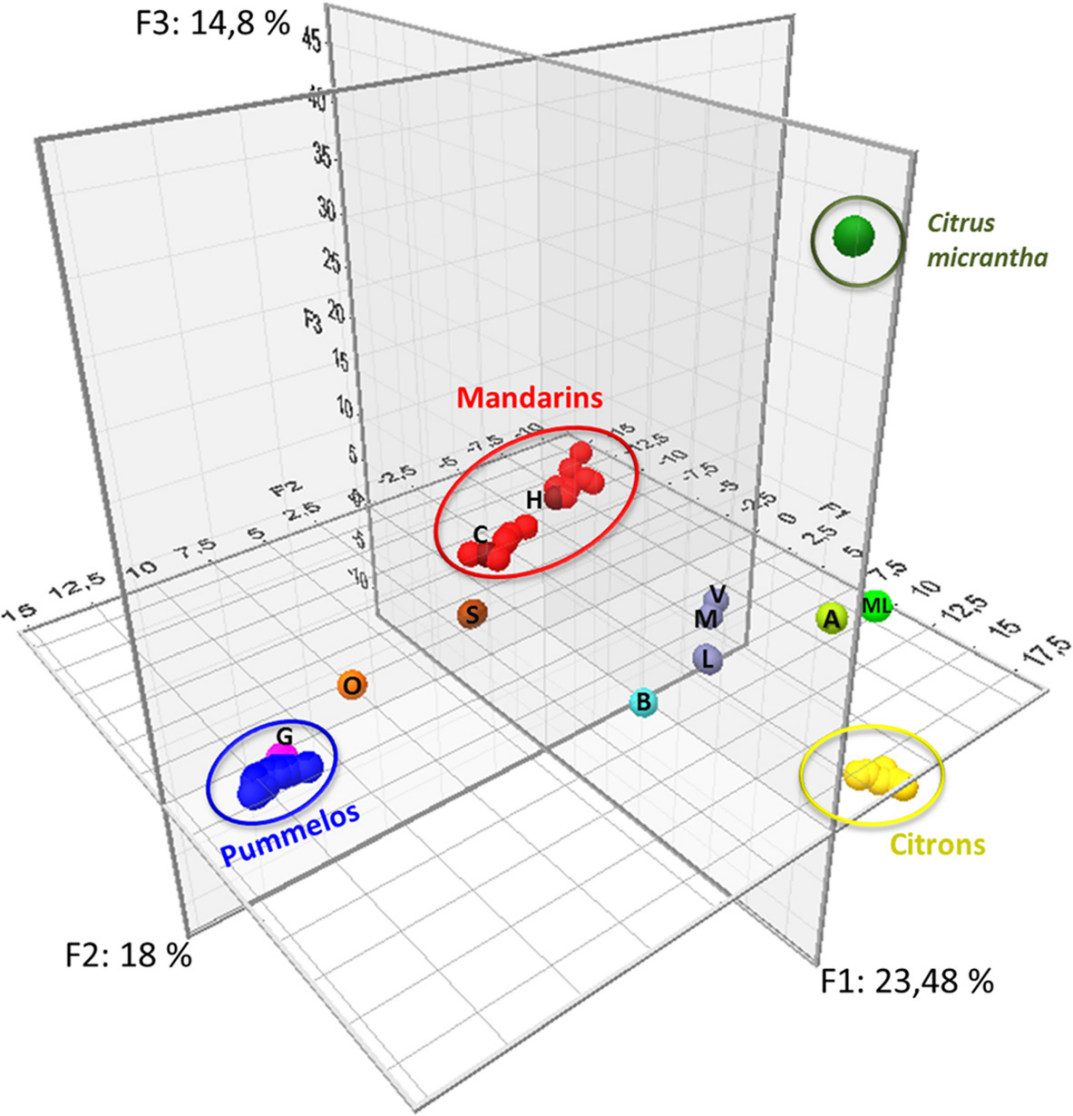
**Organization of genotypic SNP diversity.** All varieties and all SNP data were analyzed by PCA. ML: ‘Mexican’ lime; A: ‘Alemow’; V: ‘Volkamer’ lemon; M: ‘Meyer’ lemon; L: Regular and ‘Sweet’ lemons; B: Bergamot; H: Haploid clementine; C: Clementines; S: Sour oranges; O: Sweet oranges; G: Grapefruits.

PCA analysis confirmed the organization of the whole diversity coming from the four ancestral varietal groups (Figure 3). The three primary axes encompassed 56.3% of the total observed diversity. The first axis mainly separated citrons and *C. micrantha* from pummelos and mandarins. The second axis distinguished pummelos from other ancestral varietal groups. Finally, the third axis separated *C. micrantha* from other groups. ‘Alemow’ and ‘Mexican’ lime displayed intermediate positions between citrons and *C. micrantha*. Regular and ‘Sweet’ lemons and bergamot had intermediate positions between citrons and mandarins/sour oranges. Clementine lay within the mandarin cluster, while grapefruit was included in the pummelo cluster. Sweet orange and sour orange were located between the pummelo and mandarin clusters. The mandarin group displayed two noticeable subclusters. The subcluster that contained clementines and mandarins that were potentially introgressed by pummelo was displaced towards the pummelo cluster.

Analysis of linkage disequilibrium (LD) between SNPs along the chromosome [Additional file 9] also testifieds to a very high population genetic structure of the varietal sample. Significant LD values were observed across the whole chromosome, even for SNPs at distally opposing positions.

### Gene fragment haplotype inference and phylogeny

**Figure 4:**
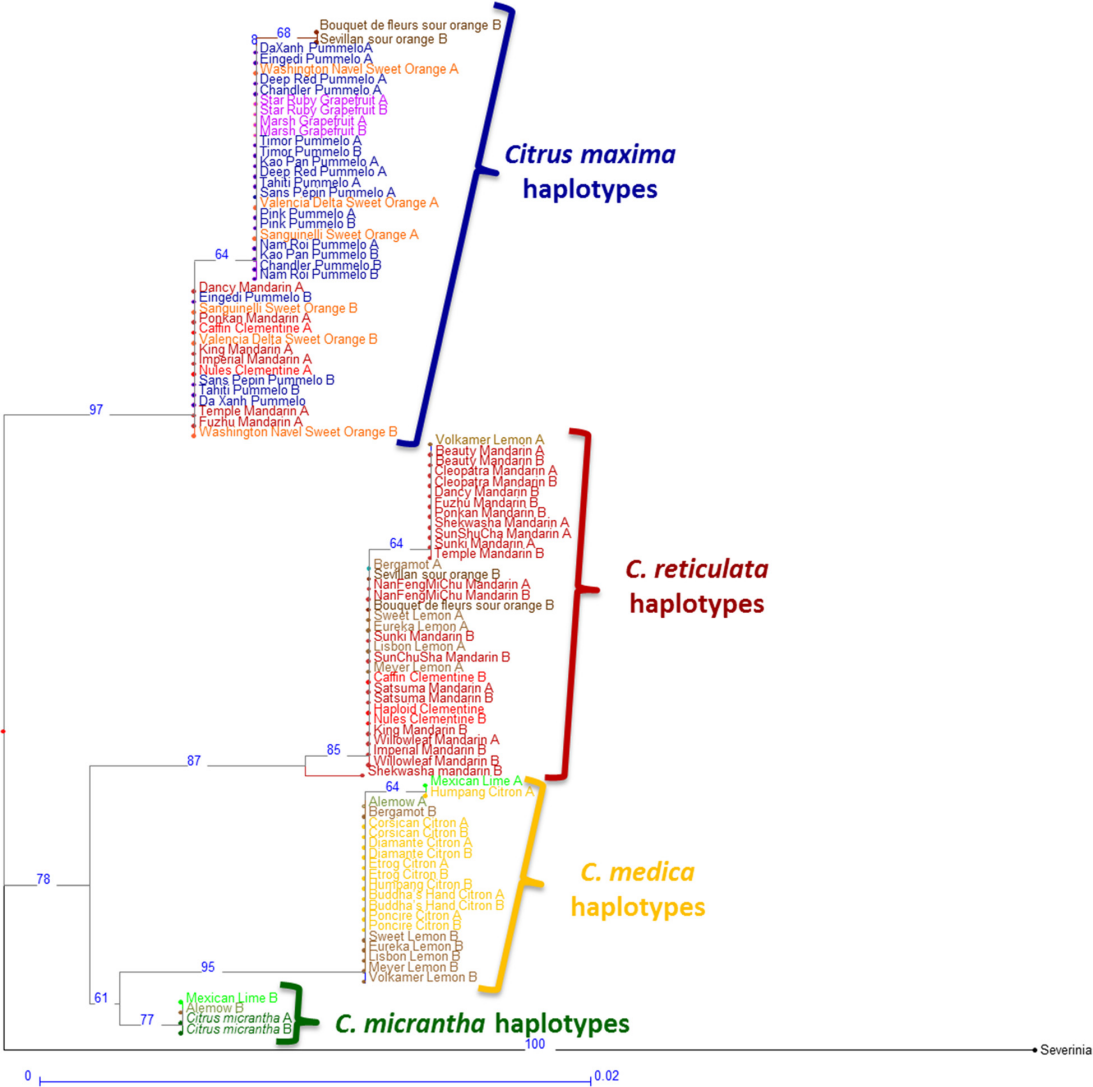
**Neighbor-joining analysis (NJA) of the haplotypic data for the 2P35391362 gene fragment.**

**Figure 5:**
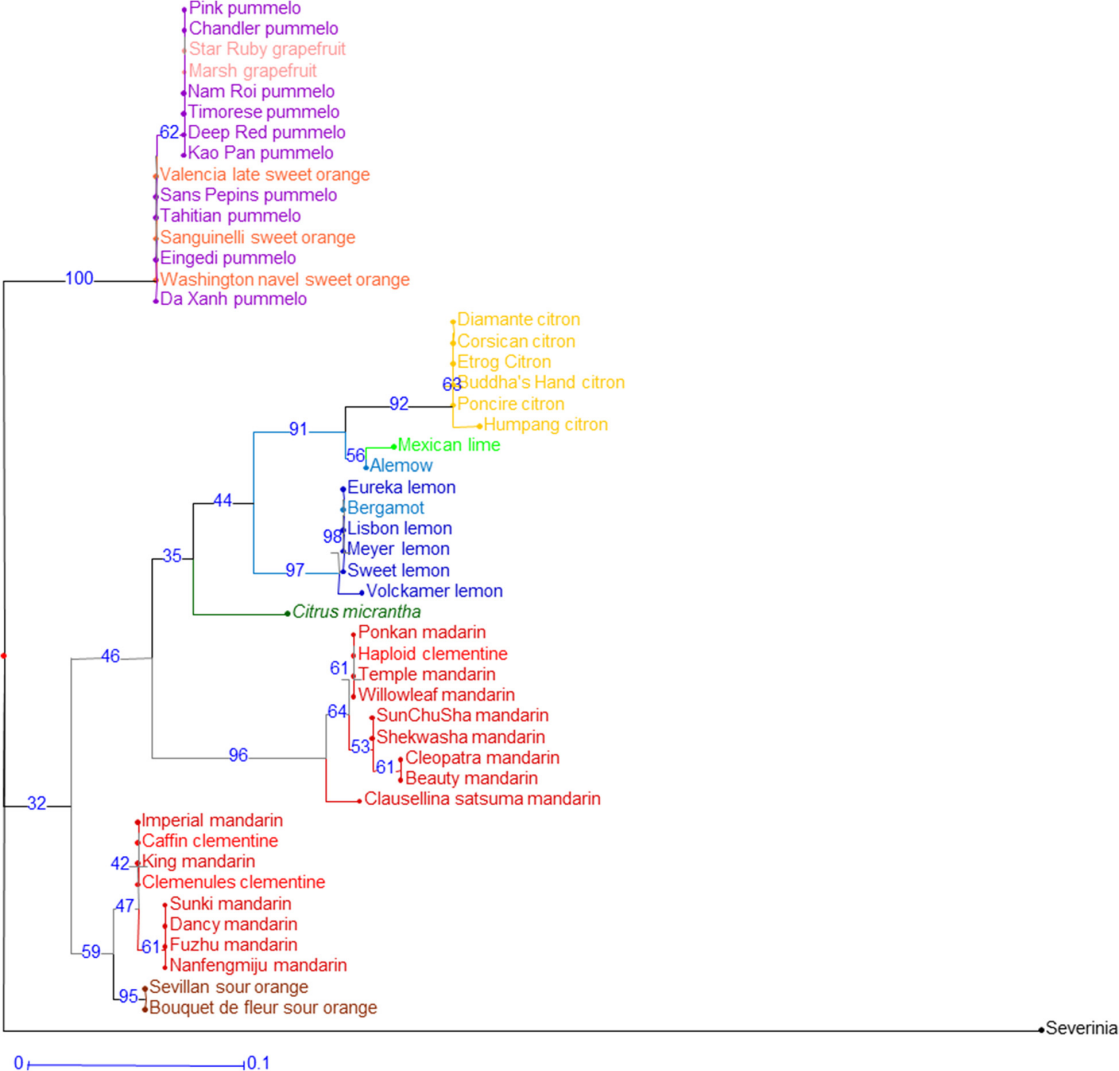
**Neighbor-joining analysis (NJA) of the genotypic data for the 2P35391362 gene fragment.**

For each gene fragment, two haplotypes were inferred for each variety. NJA and maximum likelihood analysis of haplotypes was performed to determine phylogenetic relationships, and the two methods produced the same outcomes. For example, for the 2P35391362 gene fragment (Figures 4), three, three, one, and two different haplotypes were identified in the *C. reticulata*, *C. maxima*, *C. micrantha*, and *C. medica* clusters, respectively. Multilocus haplotypic analysis also provided evidence of interspecific introgressions in varieties representative of one of the four supposed ancestral varietal groups. For this fragment, six mandarins shared one *C. maxima* haplotype with pummelos. Haplotypic analysis allowed clear inference of phylogenetic inheritance patterns for 2P35391362 in the secondary citrus species [Additional file 10]. For example, clementine clearly exhibited interspecific heterozygosity (*C. maxima*/*C. reticulata*): one haplotype was shared with sweet orange in the *C. maxima* cluster, and one was shared with ‘Willowleaf’ mandarin in the *C. reticulata* cluster. The second sweet orange haplotype was also in the *C. maxima* cluster and was shared with grapefruits that were homozygous for this haplotype. Evidence of interspecific inheritance was also found in sour orange (*C. maxima*/*C. reticulata*), bergamot (*C. medica*/*C. reticulata*), ’Eureka’, ‘Lisbon’, ‘Sweet’, ‘Volkamer’, and ‘Meyer’ lemons (*C. medica*/*C. reticulata*), and ‘Mexican’ lime and ‘Alemow’ (*C. medica*/*C. micrantha*). NJA of genotypic information from the same data set (Figure 5) provided a representation of two apparent *C. reticulata* clusters with unclear relationships. One of the clusters included accessions that exhibited interspecific inheritance when haplotype was assessed (several mandarins, sour oranges, and clementines). Similarly, lemons, limes, ‘Alemow’, and bergamot lay between *C. medica* and *C. micrantha*, clusters and branching did not provide definitive phylogenetic information.

A total of 210 haplotypes were identified through analysis of 16 gene fragments on chromosome 2 (Table 6; [Additional file 11]). From the phylogenetic analysis of each fragment, we considered 77, 58, 34, and 25 haplotypes to be representative of *C. reticulata*, *C. maxima*, *C. medica*, and *C. micrantha*, respectively. For 16 haplotypes, the organization of the genetic diversity of the corresponding fragment was insufficient to infer phylogenetic origin. The indeterminate haplotypes mostly concerned mandarin and pummelo.

**Table 6:** **Number of haplotypes attributed to the four basic taxa or with indeterminate phylogenetic origin**

**Gene fragment**	***C. reticulata***	***C. maxima***	***C. medica***	***C. micrantha***	**Indeterminate**	**Total**
2P737170	4	6	2	2	0	14
2P3068140	4	2	1	1	1	9
2P4517048	5	3	2	1	0	11
2P8108334	10	7	3	2	2	24
2P11442721	8	5	2	1	0	16
2P13928427	3	2	2	2	2	11
2P21022460	1	2	2	1	2	8
2P25198627	5	1	3	2	1	12
2P26819388	8	2	2	1	2	15
2P29538734	7	6	4	2	0	19
2P30446231	6	7	3	2	1	19
2P32507721	3	1	1	2	4	11
2P33506778	2	1	1	1	1	6
2P33532337	2	2	1	1	0	6
2P35391362	3	3	2	1	0	9
2P36235952	6	8	3	3	0	20
**Total**	**77**	**58**	**34**	**25**	**16**	**210**

**Figure 6:**
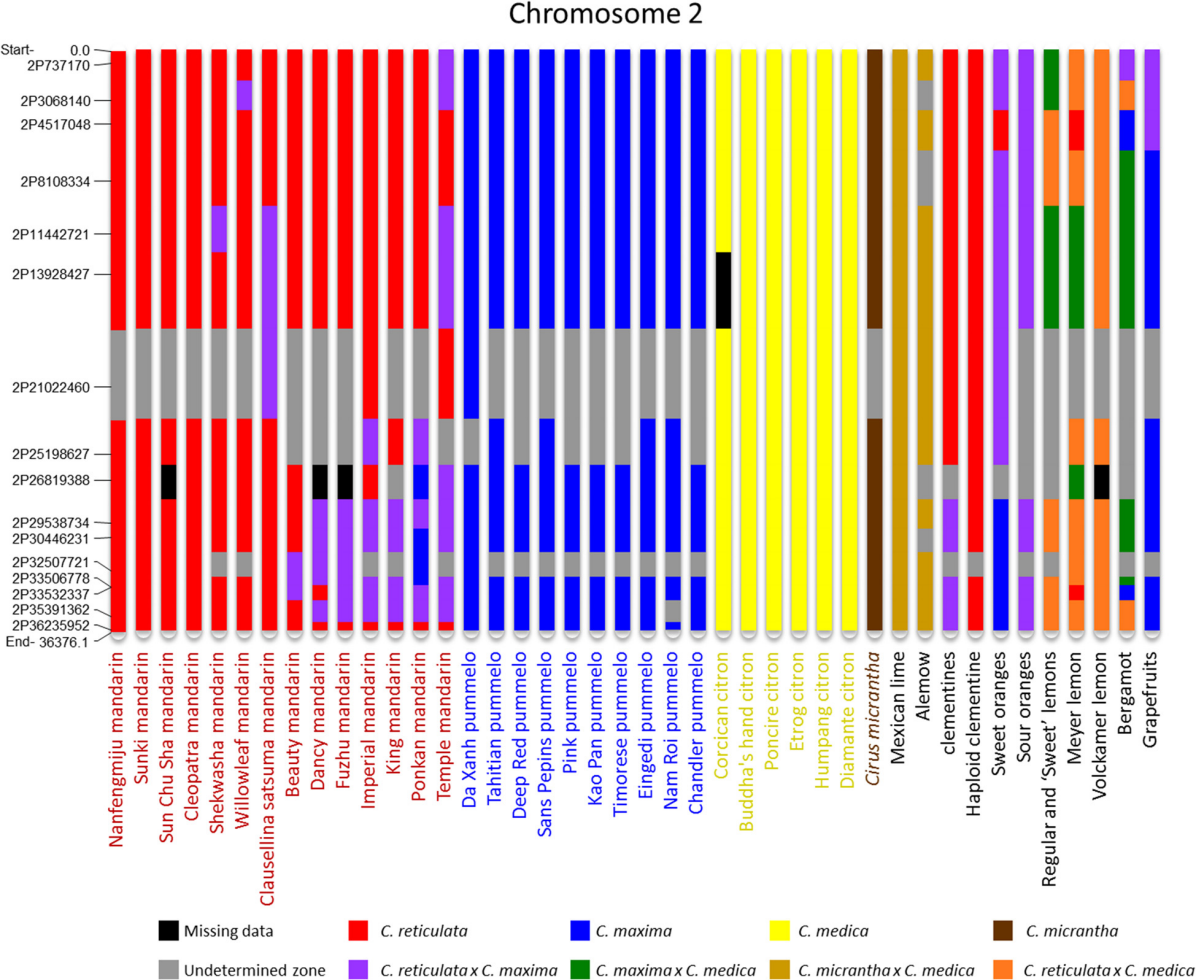
**Genotypic structure of chromosome 2 in 48** ***Citrus*** **varieties inferred from haplotypic data.**

The haplotypic structure of each accession was used to schematize the phylogenetic origin of genome fragments along chromosome 2 (Figure 6). In the absence of data regarding the phase between different haplotypes, this representation was made genotypically (homozygous for one ancestral taxon or heterozygous between two taxa). A single genotype was used to represent a varietal group when no polymorphisms were observed between varieties.

Ten of the fourteen mandarins were introgressed by *C. maxima*, mostly in heterozygosity. Two homozygous fragments for a *C. maxima* haplotype (ma1/ma1) and one fragment heterozygous for two *C. maxima* haplotypes (ma1/ma2) were found in ‘Ponkan’ mandarin. No evidence of interspecific introgression was observed for the representatives of the other three ancestral varietal groups. Completely heterozygous interspecific structures between *C. micrantha* and *C. medica* were observed for ‘Mexican’ lime and ‘Alemow’. Sour orange displayed complete heterozygosity between *C. reticulata* and *C. maxima*. Grapefruit appeared to have inherited mostly *C. maxima* haplotypes but displayed heterozygosity with *C. reticulata* at the start of the scaffold. Sweet orange was mostly heterozygous between *C. reticulata* and *C. maxima*, with a small fragment at the first part of the scaffold inherited solely from *C. reticulata*, and a genome area at the end of the scaffold inherited exclusively from *C. maxima.* Bergamot and regular, ‘Sweet’, and ‘Meyer’ lemons displayed similar structures that mainly comprised heterozygous regions of *C. medica*/*C. reticulata* and *C. medica*/*C. maxima*. However, two small homozygous regions (2P4517048 and 2P33532337 gene fragments) were observed in ‘Meyer’ lemon (*C. reticulata* homozygosity re3/re3 and re2/re2) and bergamot (*C. maxima* homozygosity ma1/ma1 and ma2/ma2). No exploitable data were obtained for one gene fragment of ‘Volkamer’ lemon. For the other 15 gene fragments, ‘Volkamer’ lemon systematically displayed one haplotype corresponding with the *C. medica* cluster. The other haplotypes for 14 of these gene fragments were assigned to the *C. reticulata* cluster. The remaining haplotype was in a cluster of indeterminate phylogeny [Additional file 12].

### Revised genetic relationships between the four basic taxa after removal of introgressed genomic regions identified in mandarin from haplotypic analysis

The identification, from haplotypic analysis, of introgressed pummelo fragments in mandarin genotypes prompted a revision of the relationships of the ancestral basic taxa (*C. maxima*, *C. reticulata*, *C. medica*, and *C. micrantha*) relative to the varietal groups deriving from these taxa (pummelos, mandarins, citrons and micrantha). The average SNP density within *C. reticulata* (Table 7) was lower (2.85 SNP/kb) than in mandarin (3.93 SNP/kb) (Table 4). Conversely, the *C. maxima*/*C. reticulata* average differentiation was 11.15 SNP/kb (10.41 SNP/kb between mandarins and pummelos). The differentiation values of *C. reticulata* with *C. micrantha* and *C. medica* were similar to those of mandarin with micrantha and citron, respectively.

**Table 7:** **Intra- and interspecies group dissimilarity (average number of SNP/kb between two varieties) after elimination of introgressed haplotypes**

	***C. reticulata***	***C. maxima***	***C. medica***
*C. reticulata*	2.85*		
*C. maxima*	11.15	1.86*	
*C. medica*	14.80	11.21	1.24*
*C. micrantha*	13.82	10.61	12.19

*Average number of SNP/kb at intra-specific level.

For each SNP, G_ST_ values were estimated for each basic species relative to all other species. This allowed estimation of the value of each considered SNP to confirm that the surrounding genome fragment was inherited from the given species (SNP specific-diagnostic points). Corrections from the introgression information increased the number of diagnostic markers for *C. reticulata* and *C. maxima* relative to the initial data for mandarin and pummelo [Additional file 13]. The number of SNP loci with an average G_ST_ value >0.8 increased from 14 and 6 for mandarins and pummelos to 27 and 10 for *C. reticulata* and *C. maxima*, respectively. The highest number of totally discriminant SNPs (G_ST_ = 1) was observed for *C. medica* (27) followed by *C. reticulata* (22), *C. micrantha* (21), and *C. maxima* (8) [Additional file 14].

## Discussion

### Genotype and haplotype information from 454 parallel sequencing of 400–600 bp amplicons can identify admixture structures and infer the evolutionary history of species with reticulate evolution

Three hundred heighten SNPs were found in 16 gene fragments from chromosome 2. The SNPs/kb rate within introns (53.6) was highly similar to the rate previously determined for the *Citrus* genus (51.5) by Garcia-Lor *et al.* [16]. The SNPs/kb rate within exons was slightly higher in this study (38.0) than in the previous study (29). Taken together, and including the small 3′ UTR regions, 48.3 SNPs/kb were identified. This rate varied between gene fragments (range: 11.2–79.7).

The observed higher heterozygosity in secondary species than in the basic taxa, as well as the higher diversity in mandarin and pummelo compared to citron, was in agreement with previous studies [15,16,18]. Moreover, the high structuration of the diversity around *C. maxima*, *C. medica*, *C. reticulata*, and *C. micrantha* revealed by Structure and PCA agreed with previous molecular [13,14,16,65] and numerical taxonomy [20] studies, which recognizes the four basic taxa as the ancestors of the cultivated *Citrus* species. The important ancestral taxon differentiation and the limited number of reticulations and further interspecific hybridizations also resulted in the generalized LD observed in this study. LD was maintained even for fragments on opposing telomeres, also noted in previous studies for markers on different chromosomes [15,18].

The relative levels of differentiation between *C. maxima*, *C. medica*, *C. reticulata*, and *C. micrantha* varied (10.61–14.8 SNPs/kb), and was on average 6.7 times higher than the within-taxon diversity (from 1.24 in *C. medica* to 2.85 in *C. reticulata*). This diversity pattern allowed inferring haplotype phylogenetic origin for 12 of the 16 genes examined on chromosome 2. Differentiation was low for the four genes in the central part of the chromosome, and this resulted in clusters of indeterminate phylogenetic origin. The indeterminate haplotypes mainly concerned mandarins, pummelos, and their secondary species haplotypes.

Haplotype analysis demonstrated *C. maxima* introgressions in genotypes generally considered to be true mandarins. After removal of these haplotypes from the analysis of the supposed ancestral taxa, higher monolocus differentiation was observed between *C. reticulata* and *C. maxima*. This also allowed more precise estimations of *C. reticulata* intraspecific polymorphism. The identification of introgressed areas from haplotypic analysis, therefore, provided better species tree reconstruction. As recommended by Ramagudu *et al.* [37], species trees can be improved by using loci that generate gene trees that are more clearly resolved. Haplotypic analysis has potential in this regard, and will allow the deselection of regions with incomplete lineage sorting or interspecific introgressions.

In the present study, 454 amplicon sequencing was successfully used to determine haplotypes in heterozygous genotypes and to analyze admixtures resulting from reticulate evolution. The broader utility of this method for identifying polymorphisms and inferring haplotype phylogenetic origins in other plants will depend on polymorphism rates within and between subspecies or species.

### Determination of the phylogenetic structure of chromosome 2 in several *Citrus* species and varieties provided insights into the origins of modern cultivated citrus

Haplotype NJA analysis of each gene fragment allowed the phylogenetic inheritance of genome fragments along chromosome 2 to be inferred for the 48 analyzed genotypes. Although a small number of haplotypes remained of indeterminate phylogenetic origin, the results provided an invaluable overview of the phylogenetic structure of chromosome 2 and the origin of modern *Citrus*.

The representative genotypes of the pummelo and citron horticultural groups appeared to be pure *C. maxima* and *C. medica*, respectively, and no interspecific introgressions were identified. Similarly, no evidence of introgression was found in *C. micrantha*. Conversely, evidence of introgression by *C. maxima* was found in 10 of the 14 mandarins studied. This corresponds with recent research [43] in which WGS analysis of ‘Willowleaf’ and ‘Ponkan’ mandarins demonstrated introgression in theses varieties considered to be true mandarins by citrus taxonomists. Three of the four mandarin varieties lacking evidence for introgression (‘Cleopatra’, ‘Sunki’, and ‘Sun Chu Sha’) are used mostly as rootstock and do not share the edible mandarin mitotype revealed by Froelicher *et al.* [66]. This particular mandarin clade should, therefore, probably not be considered as ancestral to modern cultivated mandarins. The fourth mandarin (‘Nanfengmiju’) without evidence for introgression shares the cytoplasm of edible mandarins.

The parentage hypothesis of some important commercial species and cultivars suspected to have arisen from reticulate evolution was checked by analyzing the haplotype phylogeny for each gene fragment [Additional file 10].

*Citrus sinensis* (sweet oranges) and *Citrus aurantium* (sour oranges): phenotypic data [20] and molecular marker studies [18,67,68] suggested that these two species derived from hybridizations between the *C. maxima* and *C. reticulata* gene pools. Both species have *C. maxima* maternal phylogeny as determined by chloroplast [69] and mitochondrial genome analysis [66]. In the present haplotype analysis within chromosome 2, sour orange displayed *C. maxima*/*C. reticulata* heterozygosity for each gene fragment. Sweet orange displayed *C. reticulata*/*C. reticulata* and *C. maxima*/*C. maxima* genome regions in addition to *C. maxima*/*C. reticulata* heterozygosity. The presence of a *C. maxima*/*C. maxima* region at the end of chromosome 2 disproves the hypothesis of a (*C. maxima* × *C. reticulata*) × *C. reticulata* ancestry proposed by Roose *et al.* [70] from SSR data, and Xu *et al.* [42] from WGS data. This was also determined by examination of two genes by Garcia-Lor *et al.* [16] and confirmed by whole genome resequencing data from the ICGC [43]. These results suggest a possible direct F1 interspecific origin for sour orange and a more complex origin for sweet orange that would involve two parents each with *C. reticulata* and *C. maxima* admixture. These conclusions are in agreement with those proposed by the ICGC [43]. Considering that many mandarin cultivars are introgressed by *C. maxima*, a backcross model of (pummelo × mandarin) × mandarin rather than (*C. maxima* × *C. reticulata*) × *C. reticulata* would reconcile the Wu *et al.* [43] and Xu *et al*. [42] hypotheses. For 8 of the 16 gene fragments analyzed in the present study, both sweet orange and sour orange were heterozygous but did not share haplotypes, therefore discarding the hypothesis of a direct relationship between them.

*Clementine*: It is generally agreed that, a little more than one century ago in Algeria, Father Clement selected clementine as a chance seedling from a ‘Mediterranean’ mandarin (‘Willowleaf’). Previous molecular studies suggested that clementine was a mandarin × sweet orange hybrid [13,17,18,71], and this was recently confirmed by WGS analysis [43]. From the haplotype data, the larger part of chromosome 2 in clementine appears to be inherited from *C. reticulata*, with *C. maxima*/*C. reticulata* heterozygosity at the end of the orientated chromosome (phytozome.net [46]) in agreement with WGS data [43]. The haplotype alleles of clementine, sweet orange, and ‘Willowleaf’ mandarin are in complete agreement with the hypothesis of a ‘Willowleaf’ × sweet orange origin.

*C. paradisi* (grapefruits): The origin of grapefruit is attributed to a natural hybridization between pummelo (*C. maxima*) and sweet orange (*C. sinensis*) in the Caribbean after the discovery of the New World by Christopher Columbus [15,18,72,74]. The haplotype analyses agree with this hypothesis, showing coherent haplotypes for most of the gene fragments. In grapefruit, only one fragment (2P32507721) displayed a haplotype observed neither in sweet orange nor in the pummelo accessions (nor in other basic species clusters). However, this gene fragment displayed insufficient differentiation to allow full phylogenetic assignation, and the unassigned grapefruit haplotype may have been inherited from a pummelo not included in our limited samples. Chromosome 2 of grapefruit is mainly inherited from *C. maxima* and displays a small region of *C. maxima*/*C. reticulata* heterozygosity at the start of the scaffold.

*Citrus limon* (lemons): Based on RFLP, RAPD, and CAPS data, Nicolosi *et al.* [13] proposed that “regular lemons” arose from hybridization between *C. aurantium* and *C. medica*. This hypothesis was supported by nuclear SSR [15] and SNP [18] analyses. Moreover, the maternal *C. aurantium* parentage was confirmed by study of mitochondrial indels [66]. In the present study, ‘Eureka’, ‘Lisbon’, and ‘Sweet’ lemon varieties were highly heterozygous and identical. These lemons are very likely somatic mutants of the same hybrid ancestor. The three lemons display successive genome regions with *C. reticulata*/*C. medica* or *C. maxima*/*C. medica* heterozygosities. The haplotype allele analysis completely concurs with the sour orange × citron hypothesis. Indeed, systematic haplotype sharing between lemon and sour orange and the location of the second haplotypes within *C. medica* clusters were observed. ‘Meyer’ lemon also appeared to be of tri-specific hybrid origin [15] and displayed *C. maxima*/*C. medica* and *C. reticulata*/*C. medica* heterozygosity, as well as two gene fragments homozygous for a *C. reticulata* haplotype. Even if the ‘Meyer’ lemon were found to have a sweet orange-like mitotype [66], as there were only two shared haplotypes between sweet orange and Meyer lemon over the 16 gene fragments, the haplotype analysis disproved the hypothesis that sweet orange was the female parent. ‘Volkamer’ lemon fragment gene haplotypes suggest that this genotype was a direct hybrid of *C. reticulata* and *C. medica*.

*Citrus aurantifolia* (‘Mexican’ lime, ‘Alemow’, and bergamot): These three citrus types were considered to be distinct species, namely, *C. aurantifolia*, *C. macrophylla*, and *C. bergamia* respectively, by Tanaka [10]. ‘Mexican’ lime and ‘Alemow’ displayed interspecific heterozygosity between haplotypes of the *C. medica* and the *C. micrantha* clusters. For ‘Mexican’ lime, exact haplotype sharing with the analyzed *C. micrantha* sample was found for 15 of the 16 gene fragments. This is in agreement with the hypothesis proposed by Nicolosi *et al.* [13] that suggests ‘Mexican’ lime is a *C. micrantha* × *C. medica* hybrid. Maternal phylogeny was recently confirmed by mitochondrial marker analysis [66]. Similar results were observed for ‘Alemow’. However, exact haplotype correspondence with the analyzed *C. micrantha* sample was found only for 12 gene fragments. This suggests that the maternal parent of ‘Alemow’ was closely related to the analyzed *C. micrantha*, which is in agreement with the Swingle and Reece [9] description of ‘Alemow’ as a possible hybrid of *Citrus celebica* Koord (a papeda distinct from *C. micrantha*) or some other species of the subgenus *Papeda*, with a species of the subgenus *Citrus*. In 1811, Gallesio [75] proposed that bergamot was a hybrid between lemon and sour orange. However, alternative hypotheses were proposed based on molecular studies. Chen *et al.* [76] suggested that bergamot could be a hybrid between citron and lime, Herrero *et al.* [65] and Federici *et al.* [77] proposed hybridization between sour orange and sweet lime, and hybridization between sour orange and citron was proposed by Nicolosi *et al.* [13] and Li *et al.* [78]. The present haplotypic analysis disproved the hypotheses of hybridization between sour orange and citron, and between lemon and ‘Mexican’ lime, because bergamot displayed haplotypes not found in any of these theoretical parents.

### Implications for secondary species breeding

Some secondary apomictic species such as *C. aurantium* (*C. maxima* × *C. reticulata*) and *C. aurantifolia* (*C. micrantha* × *C. medica*), or genotypes such ‘Volkamer’ lemon (*C. reticulata* × *C. medica*), displayed interspecific heterozygosity for each gene fragment. They may have resulted directly from reticulation without further sexual recombination. For such secondary species, innovative “like species” cultivars should be searched by direct hybridisation between the ancestral corresponding parental taxa, focusing on germplasm providing the suitable tolerance or resistance traits.

Conversely, other secondary species such as *C. sinensis* and *C. limon* (“Regular lemon” types) displayed more complex chromosome structures that testified to further interspecific recombination after the first reticulation events. For example, lemons (‘Eureka’, ‘Lisbon’, and ‘Sweet’ cultivars) systematically had one of their haplotypes within the *C. medica* cluster and the other in either the *C. maxima* or the *C. reticulata* cluster. Under our hypothesis of a sour orange × citron origin, the changes between C. *reticulata*/*C. medica* and *C. maxima*/*C. medica* heterozygosities along the chromosome suggest that at least three interspecific crossing over events occurred to produce the sour orange gamete that generated the lemon prototype. Previous studies [73,78] and the present work demonstrated that grapefruit resulted from hybridization between pummelo and sweet orange. For these three important citrus horticultural groups, it will be necessary to have a complete view of the nine chromosome admixture organizations to be able to rebuild similar genomic admixture structures from germplasm. Of these, “regular lemons” should be the simplest to assess despite the three-taxa structure, as it likely resulted from a relatively straightforward sequence of interspecific hybridizations (*C. maxima* × *C. reticulata*) ×*C. medica*). Genomic-assisted selection within progenies resulting from these crossing schemes should allow selection of very close interspecific mosaic structures. Such crossing will, however, be more complex for sweet orange and grapefruit because the two parents of sweet orange were themselves of interspecific origin. However, adequate pre-breeding at the parental level and genomic selection schemes over two or three generations should allow the reconstruction of similar interspecific mosaic genome structures from *C. maxima* and *C. reticulata* germplasm alongside desired resistance traits.

## Conclusion

Sixteen gene fragments on chromosome 2 were sequenced in 48 genotypes using 454 amplicon sequencing. The length of the reads and the level of differentiation between the ancestral taxa of modern citrus allowed efficient haplotype phylogenetic assignments for most gene fragments. The analysis of admixture genomic structures of modern species and cultivars revealed *C. maxima* introgressions in most modern mandarin cultivars. The haplotype results corresponded with previous hypotheses regarding the origin of many secondary citrus species, and provided a novel interpretation for the evolution of chromosome 2. Haplotyping of well-dispersed genome fragments should prove to be widely applicable, particularly for the analysis of evolutionary patterns within gene pools that experienced reticulate evolution. It is clear that this and other NGS methods will dramatically change methods of phylogenetic analysis. Regarding citrus breeding, the interspecific mosaic structure of all nine chromosome should be pursued, as this will provide the opportunity to rebuild the secondary species genomes from ancestral taxa bearing desirable traits.

## Additional files

Additional file 1:
**Excel table of varieties by common horticultural group and scientific names.**
Additional file 2:
**Excel table presenting information of amplicon location (physical and genetic), annotation of genes, and specific primers for Fluidigm amplification.**
Additional file 3:
**Excel table of multiplex genotype identifiers (MID) and related genotypes.**
Additional file 4:
**Excel table of the distribution of read numbers per gene fragment and varieties for the first Fluidigm run.**
Additional file 5:
**Excel table of the distribution of read numbers per gene fragment and varieties for the two Fluidigm runs, and solutions used for insufficient read number situations.**
Additional file 6:
**Excel table of parameters of SNP genetic diversity for each SNP position.**
Additional file 7:
**Excel table of the Heterozygosity (Ho) of secondary species.**
Additional file 8:
**Pdf document presenting the analyse of ten Structure software runs at K = 4.** Figure S1: 10 independent Structure software run clusters output permuted and aligned in order to match up as closely as possible. Table S1: Average values of the Ten Structure runs at K = 4 for each cluster of each variety (confidence interval estimated with alpha = 0.05). Additional file 9:
**Pdf document demonstrating linkage disequilibrium (LD) between SNPs on chromosome 2.**
Additional file 10:
**Pdf document demonstrating the maximum likelihood phylogenetic tree of the haplotypic data of the 2P35391362 gene fragment.**
Additional file 11:
**Pdf document demonstrating the observed inherited haplotypic structure of secondary species.**
Additional file 12:
**Excel table of the haplotypic structure of each accession.**
Additional file 13:
**Pdf document demonstrating a 3D distribution of gene sequence SNPs according to their haplotypic G**_**ST**_ **value; a: G**_**ST**_ **value for three horticultural groups (mandarins, pummelos and citrons); b G**_**ST**_ **values for three basic taxa after introgression information corrections.**
Additional file 14:
**Excel table of SNP G**_**ST**_ **values of each taxa before and after introgression information correction.**

## References

[1] FAO: **FAOSTAT** http://faostat3.fao.org/home/E**.** 2014,

[2] Ollitrault P, Navarro L, Badenes M, Byrne D (2012). Citrus. Fruit Breeding.

[3] Wang N, Trivedi P (2013). Citrus Huanglongbing: a newly relevant disease presents unprecedented challenges. Phytopathology.

[4] Grosser JW, Dutt M, Omar A, Orbovic V, Barthe G (2011). Progress towards the development of transgenic disease resistance in citrus. Acta Hort (ISHS).

[5] Texeira DC, Ayres J, Kitajima EW, Danet L, Jagoueix-Eveillard S, Saillard C, Bové JM (2005). First Report of a Huanglongbing-Like Disease of Citrus in Sao Paulo State, Brazil and Association of a New Liberibacter Species, “Candidatus Liberibacter americanus”, with the Disease. Plant Dis.

[6] Grosser JW, Deng XX, Goodrich RM: **Somaclonal variation in sweet orange: practical applications for variety improvement and possible causes.** In Citrus genetics, breeding and biotechnology. Edited by Kham IA. Wallingford: CAB International; 2007:219–233.

[7] Krueger RR, Navarro L (2007). Citrus germplasm resources. Citrus Genetics, Breeding and Biotechnology.

[8] Scora RW (1975). On the history and origin of *Citrus*. Bull Torrey Bot Club.

[9] Swingle WT, Reece PC, Reuther W, Webber HJ, Batchelor LD (1967). The botany of Citrus and its wild relatives. The citrus industry. Volume 1.

[10] Tanaka T (1961). Citologia: Semi-centennial Commemoration Papers on Citrus Studies.

[11] Mabberley DJ (1997). A classification for edible Citrus (Rutaceae). Telopea.

[12] Federici CT, Fang DQ, Scora RW, Roose ML (1998). Phylogenetic relationships within the genus *Citrus* (*Rutaceae*) and related genera as revealed by RFLP and RAPD analysis. Theor Appl Genet.

[13] Nicolosi E, Deng ZN, Gentile A, La Malfa S, Continella G, Tribulato E (2000). Citrus phylogeny and genetic origin of important species as investigated by molecular markers. Theor Appl Genet.

[14] Barkley NA, Roose ML, Krueger RR, Federici CT (2006). Assessing genetic diversity and population structure in a citrus germplasm collection utilizing simple sequence repeat markers (SSRs). Theor Appl Genet.

[15] Garcia-Lor A, Luro F, Navarro L, Ollitrault P (2012). Comparative use of InDel and SSR markers in deciphering the interspecific structure of cultivated citrus genetic diversity: a perspective for genetic association studies. Mol Genet Genomics.

[16] Garcia-Lor A, Curk F, Snoussi-Trifa H, Morillon R, Ancillo G, Luro F, Navarro L, Ollitrault P (2013). A nuclear phylogenetic analysis: SNPs, indels and SSRs deliver new insights into the relationships in the ‘true citrus fruit trees’ group (Citrinae, Rutaceae) and the origin of cultivated species. Ann Bot.

[17] Ollitrault P, Terol J, Chen C, Federici CT, Lotfy S, Hippolyte I, Ollitrault F, Berard A, Chauveau A, Cuenca J, Costantino G, Kacar Y, Mu L, Garcia-Lor A, Froelicher Y, Aleza P, Boland A, Billot C, Navarro L, Luro F, Roose ML, Gmitter FG, Talon M, Brunel D (2012). A reference genetic map of C. clementina hort. ex Tan.; citrus evolution inferences from comparative mapping. BMC Genomics.

[18] Ollitrault P, Terol J, Garcia-Lor A, Berard A, Chauveau A, Froelicher Y, Belzile C, Morillon R, Navarro L, Brunel D, Talon M (2012). SNP mining in C. clementina BAC end sequences; transferability in the Citrus genus (Rutaceae), phylogenetic inferences and perspectives for genetic mapping. BMC Genomics.

[19] Webber HJ, Reuther W, Lawton HW, Reuther W (1967). istory and development of the Citrus industry. The Citrus Industry.

[20] Barrett HC, Rhodes AM (1976). A numerical taxonomic study ofaffinity relationships in cultivated Citrus and its close relatives. Syst Bot.

[21] Ollitrault P, Jacquemond C, Dubois C, Luro F, Hamon P, Seguin M, Perrier X, Glaszmann J-C (2003). Citrus. Genetic diversity of cultivated tropical plants.

[22] Luro F, Gatto J, Costantino G, Pailly O (2011). Analysis of genetic diversity in Citrus. Plant Genetic Resources.

[23] Fanciullino AL, Dhuique-Mayer C, Luro F, Casanova J, Morillon R, Ollitrault P (2006). Carotenoid diversity in cultivated citrus is highly influenced by genetic factors. J Agric Food Chem.

[24] Stebbins G (1950). Variation and evolution in plants: Columbia University Press ed.

[25] Grant V (1981). Plant Speciation: 2nd Edit. ed.

[26] Arnold ML (1997). Natural hybridization and evolution.

[27] Doolittle WF (1999). Phylogenetic Classification and the Universal Tree. Science.

[28] Otto SP, Whitton J (2000). Polyploid incidence and evolution. Annu Rev Genet.

[29] Linder CR, Rieseberg LH (2004). Reconstructing patterns of reticulate evolution in plants. Am J Bot.

[30] Pamilo P, Nei M (1988). Relationships between gene trees and species trees. Mol Biol Evol.

[31] Rieseberg LH, Soltis DE (1991). Phylogenetic consequences of cytoplasmic gene flow in plants. Trends in Plants.

[32] Beiko RG, Hamilton N (2006). Phylogenetic identification of lateral genetic transfer events. BMC Evol Biol.

[33] Rieseberg LH, Sinervo B, Linder CR, Ungerer MC, Arias DM: **Role of gene interactions in hybrid speciation: evidence from ancient and experimental hybrids.** SCIENCE-NEW YORK THEN WASHINGTON- 1996, **272:**741–744. 10.1126/science.272.5262.741 8662570

[34] Rieseberg LH, Raymond O, Rosenthal DM, Lai Z, Livingstone K, Nakazato T, Durphy JL, Schwarzbach AE, Donovan LA, Lexer C (2003). Major ecological transitions in annual sunflowers facilitated by hybridization. Science.

[35] Rousseau-Gueutin M, Gaston A, Aïnouche A, Aïnouche ML, Olbricht K, Staudt G, Richard L, Denoyes-Rothan B (2009). Tracking the evolutionary history of polyploidy in Fragaria L. (strawberry): new insights from phylogenetic analyses of low-copy nuclear genes. Mol Phylogenet Evol.

[36] Fortune P, Pourtau N, Viron N, Ainouche M (2008). Molecular phylogeny and reticulate origins of the polyploid Bromus species from section Genea (Poaceae). Am J Bot.

[37] Ramadugu C, Pfeil BE, Keremane ML, Lee RF, Maureira-Butler IJ, Roose ML (2013). A six nuclear gene phylogeny of Citrus (Rutaceae) taking into account hybridization and lineage sorting. PLoS One.

[38] Maddison WP, Knowles LL (2006). Inferring phylogeny despite incomplete lineage sorting. Syst Biol.

[39] Jumpponen A, Jones KL (2009). Massively parallel 454-sequencing of Quercus macrocarpa phyllosphere fungal communities indicates reduced richness and diversity in urban environments. New Phytol.

[40] Sønstebø JH, Gielly L, Brysting AK, Elven R, Edwards M, Haile J, Willersleve E, Coissac E, Rioux D, Sannier J, Taberlet P, Brochmann C (2010). Using next-generation sequencing for molecular reconstruction of past Arctic vegetation and climate. Mol Ecol Resour.

[41] Taudien S, Groth M, Huse K, Petzold A, Szafranski K, Hampe J, Rosenstiel P, Schreiber S, Platzer M (2010). Haplotyping and copy number estimation of the highly polymorphic human beta-defensin locus on 8p23 by 454 amplicon sequencing. BMC Genomics.

[42] Xu Q, Chen L, Ruan X, Chen D, Zhu A, Chen C, Bertrand D, Jiao W, Hao B, Lyon PM, Chen J, Gao S, Xing F, Lan H, Chang J, Ge X, Lei Y, Hu Q, Miao Y, Wang L, Xiao S, Biswas KM, Zeng W, Guo F, Cao H, Yang X, Xu X, Cheng Y, Xu J, Liu J (2013). The draft genome of sweet orange (Citrus sinensis). Nat Genet.

[43] Wu GA, Prochnik S, Jenkins J, Salse J, Hellsten U, Murat F, Perrier X, Ruiz M, Scalabrin S, Terol J, Takita MA, Labadie K, Poulain J, Couloux A, Jabbari K, Cattonaro F, Del Fabbro C, Pinosio S, Zuccolo A, Chapman J, Grimwood J, Tadeo FR, Estornell LH, Munoz-Sanz JV, Ibanez V, Herrero-Ortega A, Aleza P, Perez-Perez J, Ramon D, Brunel D (2014). Sequencing of diverse mandarin, pummelo and orange genomes reveals complex history of admixture during citrus domestication. Nat Biotechnol.

[44] Okou DT, Steinberg KM, Middle C, Cutler DJ, Albert TJ, Zwick ME (2007). Microarray-based genomic selection for high-throughput resequencing. Nat Methods.

[45] Bybee SM, Bracken-Grissom H, Haynes BD, Hermansen RA, Byers RL, Clement MJ, Udall JA, Wilcox ER, Crandall KA (2011). Targeted amplicon sequencing (TAS): a scalable next-gen approach to multilocus, multitaxa phylogenetics. Genome Biol Evol.

[46] Department of Energy’s Joint Genome Institute, Center for Integrative Genomics: **Phytozome;** http://www.phytozome.net/**.** 2014.

[47] Fluidigm Corp.: **Access Array TM System;** http://www.fluidigm.com/access-array-system.html**;** 2014.

[48] **Curk, F.;Ancillo, G.;Garcia-Lor, A.;Luro, F.;Navarro, L.;Ollitrault, P.; Multilocus SNPs analysis allows phylogenetic assignation of DNA fragments to decipher the interspecific mosaic genome structure of cultivated citrus; Plant Genome Evolution 2011 , 4–6 Sep 2011, P2.1572** [http://f1000.com/posters/browse/summary/1089299]

[49] Life Sciences Corp. (2009). Using Multiplex Identifier (MID) Adaptors for the GS FLX Titanium Chemistry - Extended Mid Set. Technical Bulletin Genome Sequencer FLX System.

[50] Life Sciences Corp. (2009). Amplicon Fusion Primer Design Guidelines for GS FLX Titanium Series Lib-A Chemistry. Technical Bulletin Genome Sequencer FLX System.

[51] PRINSEQ: **PReprocessing and INformation of SEQuence data: Easy and rapid quality control and data preprocessing;** http://prinseq.sourceforge.net/index.html**.** 2012.

[52] Life Sciences Corp.: **454 Sequencing System Software Manual, v 2.5.3; Part C – GS De Novo Assembler, GS Reference Mapper, SFF Tools.** 454 Sequencing System Software Manual **2010:**2–213.

[53] DNASTAR Inc.: **DNASTAR Sofware for life Scientists;** http://www.dnastar.com/t-nextgen-seqman-ngen.aspx**.** 2014.

[54] Wright S (1978). Variability Within and Among Natural Populations. Evolution and the Genetics of Populations. Volume 4.

[55] Belkhir K, Borsa P, Chikhi L, Raufaste N, Bonhomme F: **GENETIX 4.05, logiciel sous Windows TM pour la génétique des populations.** *Laboratoire Génome, Populations, Interactions, CNRS UMR 5000* 1996*–*2004, (Université de Montpellier II, Montpellier (France).).

[56] Dereeper A, Nicolas S, Lecunff L, Bacilieri R, Doligez A, Peros JP, Ruiz M, This P (2011). SNiPlay: a web-based tool for detection, management and analysis of SNPs. Application to grapevine diversity projects. BMC Bioinformatics.

[57] Dereeper A, Nicolas S, Lecunff L, Bacilieri R, Doligez A, Peros JP, Ruiz M, This P: http://sniplay.cirad.fr/cgi-bin/home.cgi**.** 2013, **2014**. 10.1186/1471-2105-12-134 PMC3102043 21545712

[58] Perrier X, Jacquemoud-Collet JP: **DARwin software.** 2006, (http://darwin.cirad.fr/).

[59] Tamura K, Stecher G, Peterson D, Filipski A, Kumar S (2013). MEGA6: Molecular Evolutionary Genetics Analysis version 6.0. Mol Biol Evol.

[60] Van Berloo R (2008). GGT 2.0: versatile software for visualization and analysis of genetic data. J Hered.

[61] Pritchard Lab SU: **Structure Software;** http://pritchardlab.stanford.edu/structure.html 2014.

[62] Pritchard JK, Stephens M, Donnelly P (2000). Inference of population structure using multilocus genotype data. Genetics.

[63] Falush D, Stephens M, Pritchard JK (2003). Inference of population structure using multilocus genotype data: linked loci and correlated allele frequencies. Genetics.

[64] Evanno G, Regnaut S, Goudet J (2005). Detecting the number of clusters of individuals using the software STRUCTURE: a simulation study. Mol Ecol.

[65] Herrero R, Asins MJ, Pina JA, Carbonell EA, Navarro L (1996). Genetic diversity in the orange subfamily Aurantioideae. II. Genetic relationships among genera and species. Theor Appl Genet.

[66] Froelicher Y, Mouhaya W, Bassene JB, Costantino G, Kamiri M, Luro F, Morillon R, Ollitrault P (2011). New universal mitochondrial PCR markers reveal new information on maternal citrus phylogeny. Tree Genetics and Genomes.

[67] Uzun A, Yesiloglu T, Polat I, Aka-Kacar Y, Gulsen O, Yildirim B, Tuzcu O, Tepe S, Canan I, Anil S (2011). Evaluation of Genetic Diversity in Lemons and Some of Their Relatives Based on SRAP and SSR Markers. Plant Mol Biol Report.

[68] Uzun A, Yesiloglu T, Aka-Kacar Y, Tuzcu O, Gulsen O (2009). Genetic diversity and relationships within Citrus and related genera based on sequence related amplified polymorphism markers (SRAPs). Sci Hortic.

[69] Bayer RJ, Mabberley DJ, Morton C, Miller CH, Sharma IK, Pfeil BE, Rich S, Hitchcock R, Sykes S (2009). A molecular phylogeny of the orange subfamily(Rutaceae: Aurantioideae) using nine cpDNA sequences. Am J Bot.

[70] Roose ML, Federici CT, Mu L, Kwok K, Vu C (2009). Map-based ancestry of sweet orange and other citrus variety groups. Gentile A, Tribulato E eds Second International Citrus Biotechnology Symposium, 28 Tremestieri Etneo.

[71] Ollitrault F, Terol J, Pina JA, Navarro L, Talon M, Ollitrault P (2010). Development of SSR markers from Citrus clementina (Rutaceae) BAC end sequences and interspecific transferability in Citrus. Am J Bot.

[72] Webber HJ, Anonymous California (1943). Cultivated varieties of citrus. The Citrus Industry. History, World Distribution, Botany andVarieties.

[73] de Moraes A, dos Santos Soares Filho W, Guerra M: **Karyotype diversity and the origin of grapefruit.** Chromosome Research 2007, **15**(1):115–121. 10.1007/s10577-006-1101-2 17295131

[74] Scora RW, Kumamoto J, Soost RK, Nauer EM (1982). Contribution to the origin of the grapefruit Citrus paradisi (Rutaceae). Syst Bot.

[75] Gallesio G (1811). *Traité du citrus:* Louis Fantin ed.

[76] Chen LG, Omura M, Hidaka T (1991). A study on the taxonomy of citrus with GOT isozymes. Acta Horticulturae Sinica.

[77] Federici CT, Roose ML, Scora RW (2000). RFLP analysis of the origin of *Citrus bergamia, Citrus jambhiri*, and *Citrus limonia*. Acta Horticult.

[78] Li X, Xie R, Lu Z, Zhou Z (2010). The origin of cultivated citrus as inferred from internal transcribed spacer and chloroplast DNA sequence and amplified fragment length polymorphism fingerprints. J Am Soc Hortic Sci.

