# Supplementary material for: Next generation haplotyping to decipher nuclear genomic interspecific admixture in Citrus species: analysis of chromosome 2
Source: BMC Genet. 2014 Dec 29;15:152. doi: 10.1186/s12863-014-0152-1 (PMC4302129; doi:10.1186/s12863-014-0152-1)
Supplement: Additional file 8: — Pdf document presenting the analyse of ten Structure software runs at K = 4. Figure S1: 10 independent Structure software run clusters output permuted and aligned in order to match up as closely as possible. Table S1: Average values of the Ten Structure runs at K = 4 for each cluster of each variety (confidence interval estimated with alpha = 0.05). [file 12863_2014_152_MOESM8_ESM.pdf]

Additional file 8: analyse of 10 Structure software runs at K = 4

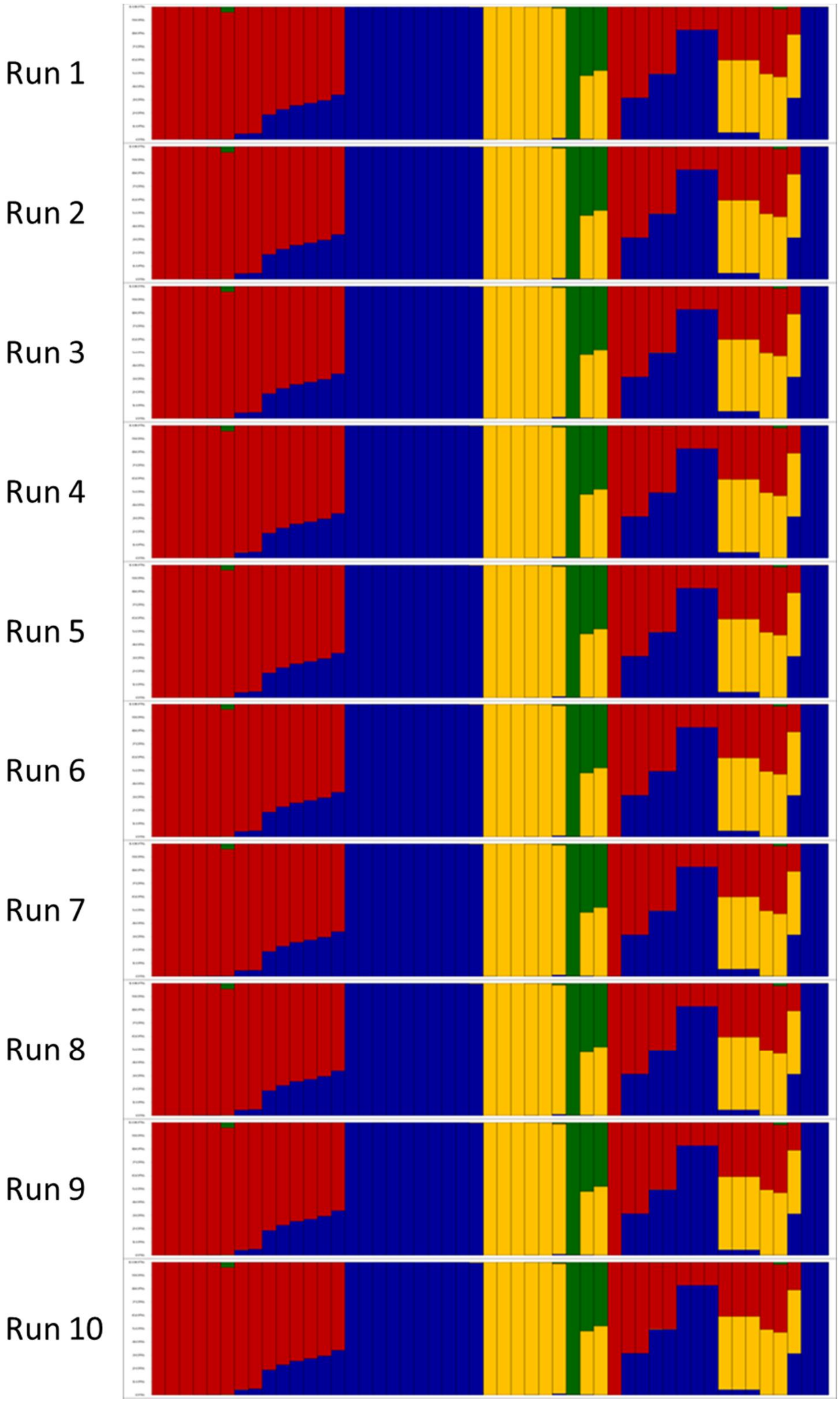

Figure Additional File 8: 10 independent Structure run clusters output permuted and aligned in order to match up as closely as possible.

Table Additional file 8: Average values of the 10 structure runs at K = 4 for each cluster of each variety (confidence interval estimated with alpha = 0.05)

	C. maxima	*C. medica*	*C. reticulata*	*C. micrantha*
Cleopatra mandarin	0.000±0.000	0.000±0.000	0.999±0.000	0.000±0.000
Sun Chu Sha mandarin	0.000±0.000	0.000±0.000	0.999±0.000	0.000±0.000
Sunki mandarin	0.000±0.000	0.000±0.000	0.999±0.000	0.000±0.000
Satsuma mandarin	0.001±0.000	0.000±0.000	0.997±0.000	0.000±0.000
Nanfengmijuu mandarin	0.001±0.000	0.001±1.343	0.994±0.000	0.003±0.000
Shekwasha mandarin	0.001±0.000	0.002±2.687	0.955±0.001	0.041±0.001
Willowleaf mandarin	0.041±0.001	0.001±0.000	0.956±0.000	0.001±1.343
Beauty mandarin	0.047±4.300	0.000±0.000	0.952±0.000	0.001±1.343
Dancy mandarin	0.187±0.000	0.000±0.000	0.811±0.000	0.000±0.000
Fuzhu mandarin	0.227±0.000	0.000±0.000	0.771±0.000	0.001±1.343
King mandarin	0.256±0.000	0.001±1.343	0.742±0.000	0.001±1.343
Temple mandarin	0.274±0.000	0.000±0.000	0.724±0.000	0.000±0.000
Imperial mandarin	0.294±0.000	0.001±1.343	0.703±0.000	0.001±1.343
Ponkan mandarin	0.336±0.000	0.000±0.000	0.662±0.000	0.001±1.343
Chandler pummelo	0.999±0.000	0.000±0.000	0.000±0.000	0.000±0.000
Da Xanh pummelo	0.999±0.000	0.000±0.000	0.000±0.000	0.000±0.000
Deep Red pummelo	0.998±0.000	0.001±1.343	0.000±0.000	0.001±1.343
Eingedi pummelo	0.999±0.000	0.000±0.000	0.000±0.000	0.000±0.000
Kao Pan pummelo	0.999±0.000	0.000±0.000	0.000±0.000	0.000±0.000
Pink pummelo	0.999±0.000	0.000±0.000	0.000±0.000	0.000±0.000
Tahitian pummelo	0.999±0.000	0.000±0.000	0.000±0.000	0.000±0.000
Timorese pummelo	0.999±0.000	0.000±0.000	0.000±0.000	0.000±0.000
Sans Pépins pummelo	0.998±0.000	0.001±1.343	0.000±0.000	0.000±0.000
Nam Roi pummelo	0.994±0.000	0.003±0.000	0.001±1.343	0.001±0.000
Buddha's hand citron	0.000±0.000	0.999±0.000	0.000±0.000	0.000±0.000
Etrog citron	0.000±0.000	0.999±0.000	0.000±0.000	0.000±0.000
Diamante citron	0.000±0.000	0.999±0.000	0.000±0.000	0.000±0.000
Poncire citron	0.001±1.343	0.998±0.000	0.000±0.000	0.001±1.343
Humpang citron	0.000±0.000	0.998±0.000	0.000±0.000	0.001±1.343
Corsican citron	0.009±0.000	0.977±0.000	0.002±0.000	0.009±0.000
Citrus micrantha	0.001±1.343	0.001±1.343	0.000±0.000	0.998±0.000
Alemow	0.004±0.000	0.476±0.000	0.001±1.343	0.518±0.000
Mexican lime	0.001±1.343	0.517±0.000	0.000±0.000	0.481±0.000
Haploid clementine	0.000±0.000	0.000±0.000	0.999±0.000	0.000±0.000
Caffin clementine	0.314±0.000	0.000±0.000	0.684±0.000	0.001±1.343
Clemenules clementine	0.314±0.000	0.000±0.000	0.684±0.000	0.001±1.343
Bouquet de fleurs sour orange	0.493±0.000	0.002±0.000	0.501±0.000	0.003±0.000
Sevillan sour orange	0.493±0.000	0.002±0.000	0.501±0.000	0.003±0.000
Sanguinelli sweet orange	0.826±6.881	0.000±0.000	0.173±1.720	0.001±1.343
Valencia late sweet orange	0.826±6.881	0.000±0.000	0.173±1.720	0.001±1.343
Washington navel sweet orange	0.825±0.000	0.000±0.000	0.173±0.000	0.001±1.343
Eureka lemon	0.046±0.003	0.547±0.001	0.404±0.001	0.001±1.343
Lisbon lemon	0.046±0.003	0.547±0.001	0.404±0.001	0.001±1.343
Sweet lemon	0.046±0.003	0.547±0.001	0.404±0.001	0.001±1.343
Meyer lemo	0.001±0.000	0.492±0.000	0.506±0.000	0.001±1.343
Volkamer lemon	0.001±1.343	0.469±0.000	0.511±0.000	0.018±0.000
Bergamot	0.313±0.000	0.477±0.000	0.208±0.000	0.001±1.343
Marsh grapefruit	0.996±0.000	0.000±0.000	0.002±0.000	0.001±1.343
Star Ruby grapefruit	0.996±0.000	0.000±0.000	0.002±0.000	0.001±1.343
