# Supplementary figures and images for: Next generation haplotyping to decipher nuclear genomic interspecific admixture in Citrus species: analysis of chromosome 2
Source: BMC Genet. 2014 Dec 29;15:152. doi: 10.1186/s12863-014-0152-1 (PMC4302129; doi:10.1186/s12863-014-0152-1)

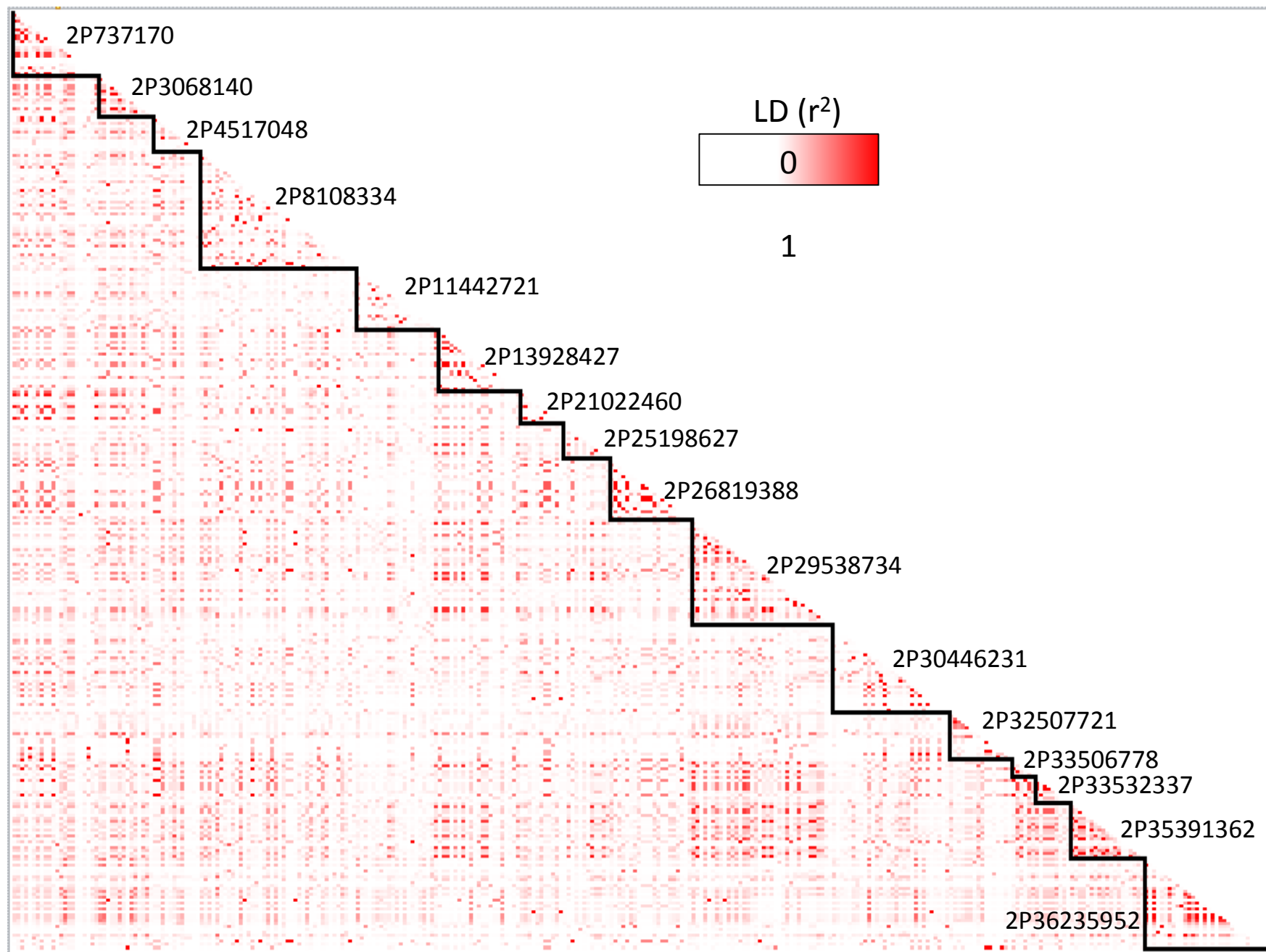

Additional file 9: Linkage disequilibrium (LD) between all SNPs along the chromosome 2

Supplement: Additional file 9: — Pdf document demonstrating linkage disequilibrium (LD) between SNPs on chromosome 2. [file 12863_2014_152_MOESM9_ESM.pdf]
