# Supplementary material for: Next generation haplotyping to decipher nuclear genomic interspecific admixture in Citrus species: analysis of chromosome 2
Source: BMC Genet. 2014 Dec 29;15:152. doi: 10.1186/s12863-014-0152-1 (PMC4302129; doi:10.1186/s12863-014-0152-1)
Supplement: Additional file 10: — Pdf document demonstrating the maximum likelihood phylogenetic tree of the haplotypic data of the 2P35391362 gene fragment. [file 12863_2014_152_MOESM10_ESM.pdf]

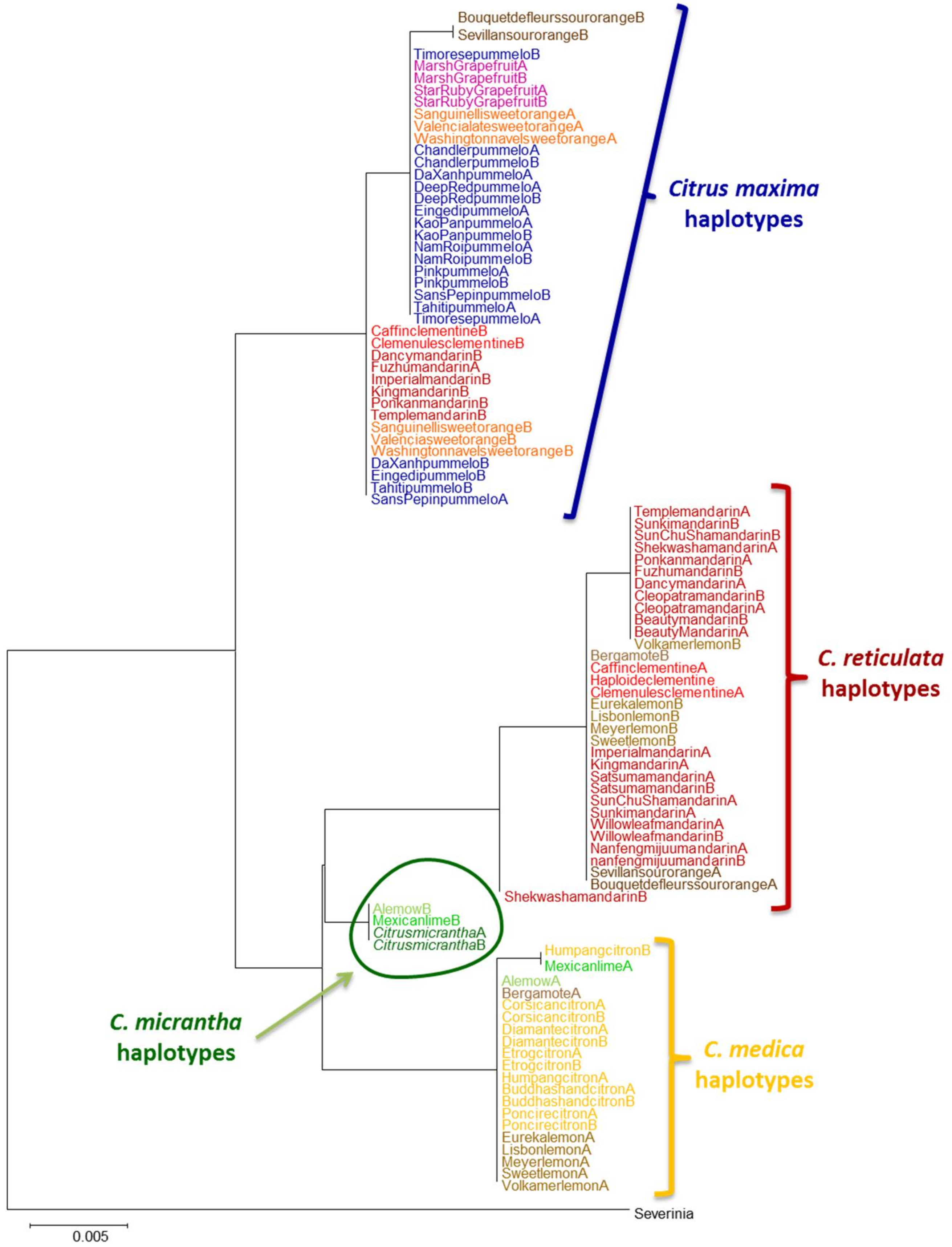

Additional File 10: Maximum likelihood phylogenetic tree of the haplotypic data of the 2P35391362 gene fragment.
