# Supplementary material for: Next generation haplotyping to decipher nuclear genomic interspecific admixture in Citrus species: analysis of chromosome 2
Source: BMC Genet. 2014 Dec 29;15:152. doi: 10.1186/s12863-014-0152-1 (PMC4302129; doi:10.1186/s12863-014-0152-1)
Supplement: Additional file 11: — Pdf document demonstrating the observed inherited haplotypic structure of secondary species. [file 12863_2014_152_MOESM11_ESM.pdf]

Additional File 11. observed inherited haplotypic structure of secondary species

	Clementine		Lemons		Mexican lime		Grapefruits		Bergamot		Alemow		Meyer lemon					
	Willowleaf mandarin haplotype	Sweet orange haplotype	Sour orange haplotype	*C. medica* haplotype	*C. medica* haplotype	*C. micrantha* haplotype	*C. maxima* haplotype	Sweet orange haplotype	Sour orange haplotype	Lemons haplotype	*C. medica* haplotype	*C. micrantha* haplotype	Sweet orange haplotype	Lemons haplotype	Sour orange haplotype	C. medica haplotype	C. reticulata haplotype	C. maxima haplotype
2P737170	✓	✓	✓	✓	✓	✓	✓	✓	✓	✓	✓	✓	✓	✓	-	✓	✓	-
2P3068140	✓	✓	✓	✓	✓	✓	✓	✓	✓	✓	✓	✓	-	✓	-	✓	✓	-
2P4517048	✓	✓	✓	✓	✓	✓	✓	✓	2	-	✓	✓	-	-	-	-	2	-
2P8108334	✓	✓	✓	✓	✓	✓	✓	✓	✓	✓	✓	-	-	-	-	✓	✓	-
2P11442721	✓	✓	✓	✓	✓	✓	✓	✓	✓	✓	✓	✓	-	✓	✓	✓	-	✓
2P13928427	✓	✓	✓	✓	✓	✓	✓	✓	✓	✓	✓	✓	-	-	-	✓	-	✓
2P21022460	✓	✓	✓	✓	✓	✓	✓	✓	✓	✓	✓	✓	-	✓	✓	✓	3	3
2P25198627	✓	✓	✓	✓	✓	✓	✓	✓	✓	✓	✓	✓	✓	✓	-	✓	✓	-
2P26819388	✓	✓	✓	✓	✓	✓	✓	✓	✓	✓	✓	1	-	-	✓	✓	-	✓
2P29538734	✓	✓	✓	✓	✓	✓	✓	✓	✓	✓	✓	✓	-	-	-	✓	✓	-
2P30446231	✓	✓	✓	✓	✓	✓	✓	✓	✓	✓	1	✓	-	-	-	✓	✓	-
2P32507721	✓	✓	✓	✓	✓	✓	1	✓	✓	✓	✓	✓	-	✓	-	✓	✓	-
2P33506778	✓	✓	✓	✓	✓	✓	✓	✓	✓	✓	✓	✓	-	✓	✓	✓	✓	-
2P33532337	✓	✓	✓	✓	✓	✓	✓	✓	2	-	✓	✓	-	-	-	-	2	-
2P35391362	✓	✓	✓	✓	✓	✓	✓	✓	✓	✓	✓	✓	-	✓	✓	✓	✓	-
2P36235952	✓	✓	✓	✓	✓	✓	✓	✓	✓	✓	✓	✓	-	✓	-	✓	✓	-
	50%	50%	50%	50%	50%	50%	47%	50%	56%	44%	44%	47%	6%	28%	16%	44%	44%	9%

1: Unidentified isolated haplotype or insufficient structuration for allowing inferring phylogenetic origin;
2: Homozygous secondary species (possible null allele); 3: missing data

Haplotype sharing between some secondary species and their supposed parents.

Hypotheses of the origin of secondary species were proposed in previous molecular studies. We have checked for each gene fragment if the haplotypic structure of these secondary species could be inherited from the supposed parents. Several molecular studies [1, 2] suggested that clementine resulted from hybridization between óWillowleafômandarin and sweet orange, regular lemons between sour orange and citron, and óMexicanô lime between *C. micrantha* and *C. medica*. These three secondary species shared 50% of their haplotypes with each of the supposed parents. Grapefruit is generally accepted as a hybrid between pummelo and sweet orange [1, 3, 4], but one of the analysed gene fragments is of unidentified origin; for all other fragments one haplotype was shared with sweet orange and the other was part of the *C. maxima* cluster. In 1811, Gallesio [5] proposed that bergamot was a hybrid between lemon and sour orange, but later molecular studies gave rise to contradictory hypotheses. For 14 gene fragments one haplotype was shared with sour orange and the other one with regular

lemon. Two gene fragments have been found to be homozygous (ma1/ma1 for the 2P4517048 gene fragment and ma2/ma2 for 2P33532337), both haplotypes shared with sour orange. It is possible that PCR competition leaded to the un-amplification of the lemon haplotype in this specific genotype. óAlemowôwas proposed to be a direct hybrid between a papeda closely related with *C. micrantha* and a citron [4]. It displayed one haplotype from the *C. micrantha* cluster and one from *C. medica* for 13 gene fragments out of 16. For five gene fragments the óAlemowôhaplotype of the *C. micrantha* cluster was different from the analysed *C. micrantha* ones. For two gene fragments one of the haplotype was in a phylogenetically undetermined cluster, and the second one was associated with the *C. medica* or *C. micrantha* clusters. For the last gene fragment it displayed only one haplotype within the *C. medica* cluster. Several origin hypotheses have been proposed for óMeyerô lemon. Chen [6], according to GOT-2 banding patterns, proposed that it was a hybrid of lime and lemon. Gulsen and Roose [7] proposed that it has a different pummelo as its female grandparent than lemon. It has also been suggested that it was a sweet orange hybrid [8]. According to our data, óMeyerôlemon is neither a sweet orange nor a sour orange hybrid. It shared *C. medica* haplotypes for 44% of the gene fragments; the two missing *C. medica* fragments correspond to a situation where óMeyerôlemon displayed homozygous haplotype. Therefore it is possible that for these fragments PCR competition leads to un-amplification of the *C. medica* haplotype, as we already supposed in the case of bergamot for the same gene fragments. In this case, the results are consistent with the hypothesis that óMeyerôlemon is a direct hybrid of *C. medica*. In this hypothesis the other parent should have an interspecific *C. reticulata* / *C. maxima* genomic structure because respectively 10 and three *C. reticulata* and *C. maxima* fragments were identified.

References

1. Nicolosi E, Deng ZN, Gentile A, La Malfa S, Continella G, Tribulato E: **Citrus phylogeny and genetic origin of important species as investigated by molecular markers.** Theoretical and Applied Genetics 2000, **100**:1155-1166.

2. Barkley NA, Roose ML, Krueger RR, Federici CT: **Assessing genetic diversity and population structure in a citrus germplasm collection utilizing simple sequence repeat markers (SSRs).** Theor Appl Genet 2006, **112**(8):1519-1531.

3. Garcia-Lor A, Curk F, Snoussi-Trifa H, Morillon R, Ancillo G, Luro F, Navarro L, Ollitrault P: **A nuclear phylogenetic analysis: SNPs, indels and SSRs deliver new insights into the relationships in the 'true citrus fruit trees' group (Citrinae, Rutaceae) and the origin of cultivated species.** Ann Bot 2013, **111**(1):1-19.

4. Ollitrault P, Navarro L: **Citrus.** In *Fruit Breeding.* Dordrecht Heildlberg edition. Edited by Badenes M, Byrne De. London: Springer New York; 2012:623-662.

5. Gallesio G: *Traité du citrus:* Louis Fantin ed. Paris: Chez Louis Fantin Libraire; 1811.

6. Chen LG, Omura M, Hidaka T: **A study on the taxonomy of citrus with GOT isozymes.** Acta Horticulturae Sinica 1991, **18**(1):27-32.

7. Gulsen O, Roose ML: **Chloroplast and nuclear genome analysis of the parentage of lemons.** J Am Soc Hort Sci 2001, **126**(2):210-215.

8. Scora RW, Kumamoto J, Soost RK, Nauer EM: **Contribution to the origin of the grapefruit Citrus paradisi (Rutaceae).** Syst Bot 1982, **7**:170-177.
