# Supplementary material for: Next generation haplotyping to decipher nuclear genomic interspecific admixture in Citrus species: analysis of chromosome 2
Source: BMC Genet. 2014 Dec 29;15:152. doi: 10.1186/s12863-014-0152-1 (PMC4302129; doi:10.1186/s12863-014-0152-1)
Supplement: Additional file 13: — Pdf document demonstrating a 3D distribution of gene sequence SNPs according to their haplotypic G ST value; a: G ST value for three horticultural groups (mandarins, pummelos and citrons); b G ST values for three basic taxa after introgression information corrections. [file 12863_2014_152_MOESM13_ESM.pdf]

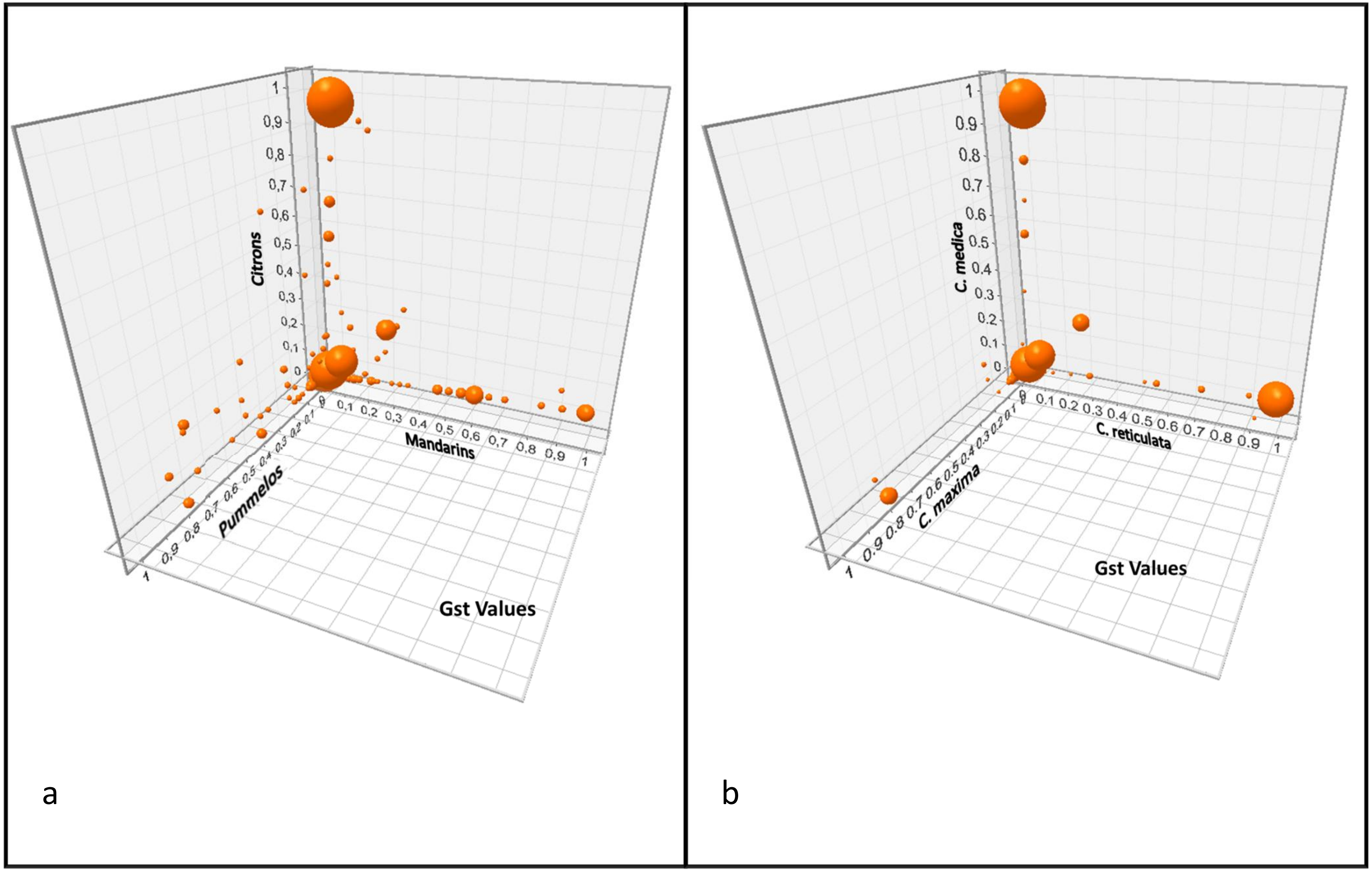

Additional file 13: 3D distribution of gene sequence SNPs according to their haplotypic G_{ST} value ; a: G_{ST} value for three horticultural groups (mandarins, pummelos and citrons); b: G_{ST} values for three basic taxa (*C. reticulata, C. maxima, C. medica*) after introgression information corrections.
